# Proteasomal-dependent CHK1 degradation leads to DNA damage accumulation in ALS cellular model systems

**DOI:** 10.1038/s41419-026-08603-6

**Published:** 2026-05-06

**Authors:** Stefania Modafferi, Valentina Silenzi, Anna Garbelli, Gloria Lazoi, Eveljn Scarian, Sara D’Uva, Tiziana Santini, Adelaide Riccardi, Mauro Cozzolino, Orietta Pansarasa, Nadia D’Ambrosi, Simone Sabbioneda, Mariangela Morlando, Sofia Francia

**Affiliations:** 1 Institute of Molecular Genetics, National Research Council (CNR), Pavia, Italy; 2 Department of Biology and Biotechnologies “Charles Darwin”, Sapienza University of Rome, Rome, Italy; 3 Cellular Models and Neuroepigenetics Section, IRCCS Mondino Foundation, Pavia, Italy; 4 Institute of Translational Pharmacology, CNR, Rome, Italy; 5 Department of Biology, University of Rome Tor Vergata, Rome, Italy

**Keywords:** Double-strand DNA breaks, Protein aggregation

## Abstract

Amyotrophic lateral sclerosis (ALS) is characterised by the aggregation of TDP-43 and mutant FUS in the cytoplasm of affected motor neurons. Accumulation of DNA damage is emerging as a novel correlative trait of ALS. We recently showed that formation of TDP-43 and FUS cytoplasmic inclusions (CIs) lead to DNA damage accumulation through dysregulation of the DNA damage response (DDR). However, the multiple molecular mechanisms contributing to DNA damage accumulation in affected motor neurons in ALS have not been fully elucidated. In recent years, chemical inhibition of the serine/threonine kinase CHK1 was shown to lead to accumulation of DNA breaks as well as increased apoptosis, in differentiated cortical neurons. Notably, CHK1 has been involved in DNA double-strand break repair in non-dividing cells, by acting through the histone chaperone ASF1A. In this article, we show that cells bearing FUS and TDP-43 CIs show downregulation of the protein levels of CHK1 and ASF1A. We observe CHK1 protein downregulation in neuronal cell lines, as well as in patient-derived motor neurons progenitors and in the spinal cord of a FUS-ALS mouse model. Restoration of the nuclear levels of CHK1 and ASF1A via transient overexpression, is sufficient to reduce DNA damage signal accumulation and rescues DDR defects*.* Importantly, we show that the ubiquitin-proteasome pathway is responsible for CHK1 degradation in cells bearing FUS CI, since its inhibition restores CHK1 and ASF1A protein levels*.* Our study demonstrates that proteasomal-dependent CHK1 and ASF1A downregulation contributes to accumulation of DNA damage in cells affected by ALS-linked protein aggregates.

## Introduction

Amyotrophic lateral sclerosis (ALS) is still an incurable progressive neurodegenerative disease caused by the death of motor neurons (MNs) and characterised by the deposition of protein aggregates inside cells of affected tissues [1]. Although the exact causes of ALS are unclear, over the years mutations in several genes have been identified. These genes encode for proteins involved in RNA metabolism, protein homeostasis, mitochondrial function, axonal transport, glutamatergic excitotoxicity and more recently also DNA repair. Consequently, these cellular pathways can be dysregulated in ALS [2]. ALS has been historically classified in familial ALS (fALS), in which there is a documented family history of ALS-associated mutations and disease onset, or sporadic ALS (sALS), in which either no mutations in ALS-linked genes have been identified in patients or mutations are de novo and no family history is observed [3].

Genes that have been found mutated in ALS patients include *TARDBP* (TAR DNA Binding Protein) and *FUS* (fused in sarcoma) that encode for the RNA and DNA binding proteins TDP-43 and FUS, respectively. In particular, *TARDBP* and *FUS* genes mutations account for about 4% fALS and 1% sALS cases [4]. TDP-43 and FUS are ubiquitously expressed, have predominantly a nuclear localization but can shuttle between the nucleus and the cytoplasm. Moreover, they share structural similarities: both possess RNA recognition motifs as well as prion-like domains that make them aggregation-prone [5]. Accordingly, these two proteins have similar functions in virtually any aspect of RNA metabolism, from mRNA splicing, stability, transport and translation to non-coding RNAs (ncRNAs) processing [6]. At the histopathological level, ALS is characterised by the presence of protein aggregates in the cytoplasm of affected MNs and glial cells. In the vast majority of cases, these protein aggregates are formed by TDP-43, in its wild-type (WT) form [3]. In contrast, FUS WT more rarely aggregates. However, frequent cytoplasmic inclusions (CIs) of FUS have been observed in samples derived from patients carrying mutations in the *FUS* gene with the mutation P525L leading to an aggressive and juvenil form of ALS [7, 8].

Accumulation of DNA damage is now emerging as a novel hallmark of ALS [9]. Accordingly, factors mutated in ALS patients have been recently involved in DNA repair, including TDP-43 and FUS [10]. The most genotoxic lesions a cell can experience are DNA double-strand breaks (DSBs). To maintain genome integrity, cells have evolved a multifarious signalling cascade that senses DSBs, transduces the signal to downstream effectors and promotes their repair. This cascade is termed DNA Damage Response (DDR) [11]. After sensing of a DSB by the DNA damage sensor MRE11-RAD50-NBS1 (MRN)-complex, the apical DDR kinase ATM (Ataxia Telangiectasia Mutated) is activated, and phosphorylates the histone variant H2AX on Ser139 in the chromatin surrounding the break. This phosphorylated form of histone H2AX is referred to as γH2AX and is a well-established marker of DSBs. The generation of γH2AX-decorated chromatin allows the recruitment of downstream DNA repair factors that ultimately leads to proper resolution of the lesion [12]. In replicating cells, DDR signalling can lead to the recruitment of BRCA1/2 (Breast Cancer Susceptibility Protein 1/2) and the DSB can be repaired by the error-free, template-based pathway of homologous recombination (HR). In contrast, in non-dividing cells, 53BP1 (P53-Binding Protein 1) is recruited, fostering repair by the most frequently used pathway of non-homologous end joining (NHEJ) [13]. Our group also discovered that the nucleases DROSHA and DICER play a role in the DDR [14, 15]. DROSHA is recruited to DSBs at the very early stages of the DDR cascade [16] and is required for the recruitment of 53BP1 [17, 18]. In ALS, TDP-43 and FUS dysfunction may lead to accumulation of DNA damage [9, 19]. Indeed, both TDP-43 and FUS interact with DROSHA and DICER during microRNA processing and ALS-associated mutations impair their roles at site of DNA damage [20–23].

The serine/threonine kinase CHK1 (Checkpoint Kinase 1) and the histone chaperone ASF1A (Anti-Silencing Function 1A) are two other important DDR factors. In replicating cells, CHK1 is activated through ATR (Ataxia Telangiectasia and Rad3-related)-mediated phosphorylation in response to the accumulation of single-stranded DNA (ssDNA) generated by replication stress [24]. Several lines of evidence also support the role of CHK1 and ASF1A in DSB repair. In particular, it was found that the key HR factor RAD51 is a substrate of CHK1 phosphorylation, and loss of CHK1 impedes RAD51 foci formation [25]. In NHEJ, ATM-dependent CHK1 activation allows phosphorylation of ASF1A, promoting 53BP1 focal recruitment and DNA repair [26]. ASF1 seems to promote NHEJ also by limiting DNA end resection [27, 28]. Moreover, CHK1 inhibition can cause DSB accumulation and cell death in several cancer cells and in fact CHK1 inhibitors are under trial to induce apoptosis in cancer [29].

Given that ALS is caused by the deposition of insoluble protein aggregates, is often considered a proteinopathy [1]. Hence, pathways that maintain protein homeostasis are particularly relevant in ALS. The main pathways responsible for protein degradation are the ubiquitin-proteasome system (UPS) and autophagy. In UPS-mediated protein degradation, unfolded proteins or polypeptides are conjugated to ubiquitin chains and subsequently cleaved into peptides by passing through the barrel-like proteasome, composed of subunits with protease activity. In contrast, during autophagy, soluble or aggregated proteins, as well as damaged organelles, are engulfed in single- or double-membrane vesicles and targeted to lysosomes [30]. Several mutations in UPS and autophagy components have been identified in ALS patients. These mutations decrease their ability to clear aberrant proteins, thus favouring their accumulation thus likely their aggregation [31].

In this study, we investigated whether the loss of CHK1 and ASF1A may trigger the accumulation of DNA damage of cells bearing TDP-43 and FUS CIs that we observed in our previous work [32]. We found that CHK1 and ASF1A protein levels are reduced in the nucleus of cells in different ALS cellular model systems, as well as in the spinal cord tissue of an in vivo FUS mouse model. Importantly, we found that the restoration of CHK1 and ASF1A nuclear protein levels, by inhibition of proteasomal degradation is sufficient to rescue the focal recruitment of DDR factors and prevent DNA damage accumulation in cells bearing CIs.

## Materials and methods

### Cell culture and treatments

Cell lines were authenticated by STR profiling (GenePrint system, Promega) and tested for mycoplasma contamination. HeLa (ATCC) and HT-22 cells were cultured in Dulbecco’s Modified Eagle Medium (DMEM) supplemented with 1% L-glutamine, 1% penicillin/streptomycin, and 10% foetal bovine serum (FBS). Where indicated cells were treated with 50 ng/mL neocarzinostatin (NCS) (Sigma, #N9162) for 20 min at 37 °C to induce formation of DSBs. For autophagy inhibition, cells were treated with 100 nM bafilomycin A1 (BafA1) (Selleckchem, #S1413) dissolved in DMSO for 20 h at 37 °C. For proteasome inhibition in cells overexpressing mutant FUS, cells were treated with 5 µM MG132 (Millipore, #474787-10MG) dissolved in DMSO for 20 h. For proteasome inhibition in untransfected HeLa cells, cells were treated with 5 µM MG132 for 24 and 48 h. To inhibit chaperone-mediated autophagy, cells were treated with 20 µM VER-15508 (Sigma Aldrich, #SML0271-5MG) 48 hours prior to plasmid transfection. The inhibitor was left in the culture media until the end of the experiment (for a total 72 h of treatment). Human motor neuron progenitors (hMNPs) were derived from a control individual or a sALS patient. Briefly, peripheral blood mononuclear cells (PBMCs) were collected from a sALS patient or a healthy individual. Next, they were reprogrammed into induced pluripotent stem cells and differentiated first into neural stem cells and then in MNPs [33]. Mouse embryonic stem cells (mESCs) were cultured and differentiated into spinal MNs as described in [34].

### Plasmid and siRNA transfection

To generate the FUS split system, WT or P525L FUS were cloned in pCS2plus-mCerulean 156-239-GGGS (Addgene, #162616) and pCS2plus-mVenus1-155-GGGS (Addgene, #162610) [32, 35]. When transfecting FUS split systems vectors, 0.7 µg of each vector were transfected using Lipofectamine^TM^ 2000 (Invitrogen, #11668-019) according to the manufacturer’s instructions. pCS2plus-mCerulean 156-239-GGGS and pCS2plus-mVenus1-155-GGGS were a gift from Marco Morsch [35]. To overexpress CHK1, 0.5 µg of FUS P525L mVenus, 0.5 µg of FUS P525L mCerulean and 1 µg of pcDNA4-Chk1-Flag (Addgene, #22894, a gift from Aziz Sancar laboratory [36] or 1 µg of pcDNA3.1+ (Thermo Fisher Scientific) (used as an empty vector (EV)) were transfected in each well. For overexpression of FUS P525L and ASF1A, 0.5 µg of FUS P525L mVenus, 0.5 µg of FUS P525L mCerulean and 1.5 µg of ASF1A Human Tagged ORF Clone (ORIGENE, #RC200324) or 1.5 µg of EV were transfected in each well. For sorting, cells were seeded in one 60 mm dish per condition. Transfection reactions were scaled up doubling every volume. In this case, pCS2plus-mCerulean 156-239-GGGS was used as EV because transfection of both pCS2plus-mCerulean 156-239-GGGS and pCS2plus-mVenus1-155-GGGS results in virtually all cells becoming positive, thus preventing us from being able to sort cells. For overexpression of WT and mutant TDP-43, 1 µg of each plasmid was transfected as described above. The WT TDP-43-encoding vector [37] was a kind gift of Dr. Emanuele Buratti on concession of Dr. Leonard Petrucelli (Mayo Clinic, Jacksonville, Florida), the plasmid expressing TDP-43 M337V was kindly provided by Prof. Eran Hornstein on concession of Prof. Dr. Markus Landthaler (Max Delbrück Center for Molecular Medicine-MDC, Berlin, Germany) whereas the plasmids encoding for A382T TDP-43 and I383V TDP-43 were generated in house via single nucleotide mutation of the plasmid expressing WT-TDP-43. For overexpression of CHK1 and WT TDP-43, 0.5 μg of WT TDP-43 and 0.5 μg of pcDNA4-Chk1-Flag or 0.5 μg of EV were transfected in each well as described above. For overexpression of ASF1A and WT TDP-43, 0.5 μg of WT TDP-43 and 1.5 μg of ASF1A or 1.5 μg of EV were transfected in each well as described above. For RNA interference, siRNAs were transfected using Lipofectamine RNAiMax^TM^ (Invitrogen, #13778-075) together with P525L FUS plasmid transfection according to the manufacturer’s instructions. The following siRNAs were used in this study: 20 nM siCTRL (ON-TARGETplus Non-targeting siRNA #1, Dharmacon, #D-001810-01-20), siCHK1 (10 nM Hs_CHEK1_7 FlexiTube siRNA, Qiagen, #SI00299859; 10 nM Hs_CHEK1_8 FlexiTube siRNA, Qiagen, #SI00605094) in CHK1 knockdown experiments, and 20 nM siCTRL (ON-TARGETplus Non-targeting Control Pool, Dharmacon, #D-001810-10-20), siTRA2A/B (10 nM ON-TARGETplus Human TRA2A siRNA, Dharmacon, #L-019480-00-0005; 10 nM ON-TARGETplus Human TRA2B siRNA, Dharmacon, #L-007278-00-0005) in TRA2A/B knockdown experiments.

### Indirect Immunofluorescence (IF) and image acquisition

In HeLa and HT-22 cells, IF was performed as previously described in [32]. In hMNPs, IF was performed as described in [33]. Briefly, coverslips were fixed in 4% PFA for 15 minutes at RT, washed twice in 1x PBS and permeabilised in 0.2% Triton for 10 minutes at RT. For blocking, coverslips were incubated in 1x PBG (0.5% bovine serum albumin -BSA-, 0.2% gelatine from cold water fish skin (Sigma Aldrich, #G7765) in 1x PBS) for one hour at RT. Primary antibodies mixes were prepared in 1x PBG and incubated overnight at 4°C in a humid chamber. The next day IF was performed as described in [32]. mMNs were cultured for 2 days after embryoid body dissociation and were fixed in 4% PFA (Electron Microscopy Sciences)/4% sucrose/5 mM MgCl_2_/1x PBS for 30 min at 4 °C. Cells were then washed twice with 4% sucrose/1x PBS. Following the dehydration steps with room temperature ethanol (50%, 70%), for short-term storage, cells were stored at 4 °C in 70% ethanol. Cells were rehydrated with 50% ethanol followed by a 10 min incubation with 1x PBS. Cells were permeabilized with 0.25% Triton X-100/1x PBS for 20 min at RT. For blocking, coverslips were incubated in 2.5% donkey/2.5% goat serum/1x PBS for 45 min at RT. Anti-CHK1 (1:200) and anti-beta III tubulin (1:200) primary antibodies were incubated overnight at 4 °C. Donkey anti-mouse Alexa Fluor Plus 647 and donkey anti-chicken Alexa Fluor 488 secondary antibodies were diluted 1:200 in 2.5% donkey/2.5% goat serum/1x PBS and incubated for one hour at RT. For a complete list of the antibodies used in this study see Table 1. Images were acquired using a confocal linear laser-scanning microscope (Zeiss LSM 800) equipped with four lasers [diode laser 405 nm (5 mW), diode laser 488 nm (10 mW), diode laser 561 nm (10 mW), and diode laser 640 nm (5 mW)], two Master gain with high sensitivity, and a 63×/1.4NA objective. This microscope was driven by the Zeiss Zen Blue 2.6 software. To quantify the number of foci per nucleus or the mean nuclear intensity for each marker, the CellProfiler v2.1.1 software [38]. was used. This software allows the user to set pipelines that enable objective, automated, and reproducible quantification of cell phenotypes. The mean nuclear intensity was normalised on the control condition (EV or vehicle-treated) using Microsoft Excel.

**Table 1:** List of antibodies used in this study.

Target	Host	Dilution	Application	Manufacturer	Cat. N.
Primary Antibodies					
53BP1	goat	1:1000	IF	Bethyl	A303-906A
53BP1	rabbit	1:1000	IF	Bethyl	A300-272A
ASF1A	rabbit	1:300	IF	Cell Signaling	2990
CHK1	mouse	1:500	IF	Cell Signaling	2360
CHK1	mouse	1:200	IF mMNs	Cell Signaling	2360
CHK1	rabbit	1:10 000	WB	Novus	NB100-464
DROSHA	rabbit	1:500	IF	Abcam	ab183732
FK2	mouse	1:1000	WB	Enzo Life Sciences	ENZ-ABS840
FLAG	mouse	1:800	IF	Sigma	F1804
FLAG	rabbit	1:800	IF	Cell Signaling	14793S
FLAG	rabbit	1:1000	WB	Cell Signaling	2368S
FUS	rabbit	1:2000	WB	Bethyl	A300-293A
GAPDH	mouse	1:1000	WB	Abcam	ab8245
H3	mouse	1:2000	WB	Active Motif	39064
p62	rabbit	1:1000	WB	Enzo Life Sciences	BML-PW9860
TDP-43	goat	1: 200	IF	Arigo	ARG64788
TDP-43	mouse	1:100	IF	Abcam	ab57105
TIA-1	rabbit	1:300	IF	Abcam	ab40693
VINCULIN	mouse	1:2000	WB	Millipore	MAB3574
β-tubulin III	chicken	1:200	IF mMNs	Sigma Aldrich	AB9354
γH2AX	mouse	1:1000	IF	Millipore	05-636
γH2AX	rabbit	1:500	IF	Cell Signaling	9718
Secondary antibodies					
anti-chicken Alexa Fluor 488 IgY	donkey	1:200	IF mMNs	Sigma Aldrich	SAB4600031
anti-goat Alexa Fluor 488 IgG	donkey	1:400	IF	Abcam	ab150129
anti-goat Alexa Fluor 647 IgG	donkey	1:400	IF	Abcam	ab150131
anti-goat Alexa Fluor 555 IgG	donkey	1:400	IF	Invitrogen	A21432
anti-mouse Alexa Fluor 647 IgG	donkey	1:400	IF	Abcam	ab150107
anti-mouse Alexa Fluor 488 IgG	donkey	1:400	IF	Abcam	ab150105
anti-mouse Alexa Fluor 555 IgG	donkey	1:400	IF	Invitrogen	A31570
anti-mouse Alexa Fluor 647 IgG	donkey	1:200	IF mMNs	Thermo Fisher Scientific	A32787
anti-mouse IgG HRP	donkey	1:10 000	WB	Abcam	ab97030
anti-rabbit Alexa Fluor 488 IgG	donkey	1:400	IF	Abcam	ab150073
anti-rabbit Alexa Fluor 647 IgG	donkey	1:400	IF	Abcam	ab150075
anti-rabbit Alexa Fluor 555 IgG	donkey	1:400	IF	Invitrogen	A31572
anti-rabbit IgG HRP	donkey	1:10 000	WB	Abcam	ab97064

### Cell sorting

Plasmids encoding for either WT or mutant P525L FUS fused with either the N-terminal YFP protein fragment mVenus (mVen) or the CFP C-terminal protein fragment mCerulean (mCer) were co-transfected. Upon natural interaction between two FUS proteins, the fluorescent protein is reconstituted, emitting fluorescence in the nucleus in cells overexpressing WT FUS or fluorescent CIs in cells overexpressing the mutant protein. The day after plasmid transfection, cells were washed twice with 1x PBS, gently scraped and collected in 1 mL of sorting buffer (2 mM EDTA, 20 mM HEPES in distilled water pH 7.3 in 1x PBS) per condition, collected in 15 mL tubes, and placed in ice. Next, samples were transferred onto a 5 mL falcon equipped with a 35 µm nylon mesh (Corning, #3532235) and vortexed thoroughly to dissociate any cell clusters that might have formed. Finally, samples were analysed on an S3e Cell sorter (BioRad). First, 30 000 cells transfected with the EV were analysed to set the gates. Next, cells overexpressing WT FUS were sorted in purity mode to obtain a pure population of cells with fluorescent nuclei, followed by sorting of cells overexpressing P525L FUS in purity mode to isolate a pure population of cells bearing fluorescent CIs. Hence, analysis of *CHK1* and *ASF1A* mRNA levels and *CHK1* alternative splicing was conducted in pure populations of cells overexpressing either WT FUS (in the nucleus) or mutant FUS (CIs in the cytoplasm). Following cell sorting, cells were centrifuged at 4 °C for 1.5 min at full speed, and the supernatant was discarded. At this point, cells were either lysed for RNA extraction or frozen at −80 °C for future use.

### Western blotting

Cells were lysed in an appropriate volume of 1x Laemmli buffer (2% SDS, 5% glycerol, 60 mM Tris-HCl pH 6.8 in deionised water) by scraping, boiled for 5 minutes at 95 °C and syringed until they were liquid. For extraction of proteins from murine spinal cord samples, about 25 mg of tissue were lysed in 250 µL of Laemmli buffer containing 1x protease inhibitor. Each sample was then smashed with potters and incubated in ice for 30 min. After incubation, they were syringed and centrifuged at 1300 rpm for 10 minutes at 4 °C. Next, the supernatant was collected and quantified. The obtained protein lysates were quantified using the Pierce BCA Protein Assay Kit (Thermo Scientific, #23227) according to the manufacturer’s instructions. After quantification, 25 µg of protein lysates were mixed with 0.1 M DTT and 0.01% bromophenol blue, boiled for 5 minutes at 95 °C and loaded onto a mini-PROTEAN TGX stain-free gel (Biorad, #4568095), together with 10 µL of Precision Plus Protein^TM^ Standard (Biorad, #161-0374). The gel was run at 60 V and 100 V for the stacking and resolving gel, respectively, in 1x Tris/Glycin/SDS (TGS) buffer (25 mM Tris, 250 mM glycine and 0.1% SDS). Next, proteins were transferred on a nitrocellulose membrane (Trans-Blot® Turbo Transfer Pack, Biorad, #1704158) using the Trans-Blot Turbo Transfer System v1.02 (Biorad) with a voltage of 25 V for 12 minutes. Blocking was performed in 5% skim milk in 1x TBS-T (TBS buffer (10 mM TRIS-HCl 1 M pH 7.5, 150 mM NaCl) + 0.1% Tween-20) for one hour at RT while shaking. Primary antibodies were prepared in 5% skim milk and incubated overnight at 4 °C while shaking. The next day, membranes were washed three times in TBS-T for 5 minutes each time and incubated with secondary antibodies prepared in 5% skim milk in 1x TBS-T for one hour at RT while shaking. After secondary antibody incubation, membranes were washed three times in 1x TBS-T for 15 minutes each time while shaking and incubated with the HRP substrate (Luminata Classico (Millipore, #WBLUC0500) or Luminata Crescendo (Millipore, #WBLUR0500)) for two minutes at RT in the dark. Membranes were finally developed using a ChemiDocTM MP Imaging System (Biorad). For a complete list of the antibodies used in this study see Table 1. Densitometric analysis was performed using the ImageLab (Biorad) software.

### RT-qPCR

Total RNA extraction was performed using the RNeasy Mini Kit (Qiagen, #74106) according to the manufacturer’s instructions. Genomic DNA removal was performed using the TURBO DNA-free kit (Invitrogen, #AM1907) according to the manufacturer’s instructions. After extraction, RNA purity and concentration were determined using Nanodrop (Thermo Fisher Scientific). For reverse transcription, 1 µg of RNA was retrotranscribed using the SuperScript IV First-Strand Synthesis System (Invitrogen, #18091050) according to the manufacturer’s instructions. Next, 15 ng of cDNA were used for RT-qPCR with QuantiTect SYBR® Green (Qiagen, #204145) and a LightCycler® 480 II Sequence Detection System (Roche). The following thermal protocol was used: denaturation: 95 °C for 15 minutes (one cycle), denaturation/annealing/extension: 95 °C for 15 seconds > 60 °C for 20 seconds > 72 °C for 30 seconds (45 cycles); melting curve: 40 °C > 90°C > 40°C. For hMNPs, RNA extraction was performed as described in [33]. and 500 ng of RNA were reverse-transcribed as described above. mMN total RNA extraction was performed using the Direct-zol Miniprep RNA Purification Kit (Zymo Research) with an on-column DNase treatment, according to the manufacturer’s instructions. For samples derived from the spinal cord of FUS-ALS mice, total RNA was extracted from mice lumbar spinal cord using Trizol reagent (Thermo Fisher Scientific, #15596026), followed by DNase I treatment (Promega, #M6101), according to manufacturers’ instructions. RNA from mice and mMNs was reverse-transcribed using the PrimeScriptTM RT Reagent Kit (Takara Bio, #RR037B), according to the manufacturer’s instructions. cDNA samples (10 ng/reaction) were analyzed by RT-qPCR using PowerUp SYBR Green Master Mix (Life Technologies, #A25742), according to the manufacturer’s protocol, on a QuantStudio 3 Real-Time PCR (Thermo Fisher Scientific). RNA levels are relative to GAPDH mRNA, used as a control gene. Relative RNA quantity was calculated as the fold change (2-ΔΔCt) with respect to the control samples set as 1. For the complete list of primers used in this study see Table 2.

**Table 2:** List of primers used in this study.

Gene	5’-3’ sequence
hCHK1_tot_FW	CAACAAACCCCTCAAGAAAGG
hCHK1_tot_REV	TGGATTGAATGTGCTTAGAAAATC
hASF1A_tot _FW	CAGATGCAGATGCAGTAGGC
hASF1A_tot _REV	CCTGGGATTAGATGCCAAAA
mChk1_tot_FW	GCCCAGTGATAGCTGTCAGG
mChk1_tot_REV	GGCCTCTTTGCTCCTCTGTT
mChk1_exon2_FW	TCGCTGTGCTTGGAGTCATGGC
mChk1_exon4_REV	GAACCTCTGAGCATCTTGTTCAGG
hCHK1_exon3_FW	GACTGGGACTTGGTGCAAAC
hCHK1_exon3_REV	TGCCATGAGTTGATGGAAGA
mGapdh_FW	AATGGTGAAGGTCGGTGTGAAC
mGapdh_REV	CCGTGAGTGGAGTCATACTGGAAC
RPLP0_FW	ATGCCCAGGGAAGACAGGGCG
RPLP0_REV	CGAAGGGACATGCGGATCTGCTGC
hGAPDH_FW	GGAAGGTGAAGGTCGGAGTC
hGAPDH_REV	TTACCAGAGTTAAAAGCAGCCC

### Alternative splicing analysis

For splicing analysis in HeLa and hMNPs, the GoTaq® G2 Flexi DNA Polymerase (Promega, #M7805) was used according to the manufacturer’s instructions using 1 ng/µL of cDNA as template. For HeLa cells, cDNA was synthesized from pure cell populations derived from cell sorting. For analysis of CHK1 exon 3 alternative splicing, 0.2 µM of each gene-specific primer were used with the following thermal protocol: initial denaturation: 95 °C for 2 min (one cycle), denaturation/annealing/extension: 95 °C for 15 seconds > 50 °C for 20 seconds > 74 °C for 20 seconds (35 cycles), final extension: 74 °C for 5 min (one cycle). For GAPDH amplification, 1 µM of each gene-specific primer was used with the following thermal protocol: initial denaturation: 95 °C for 1:50 minutes (one cycle), denaturation/annealing/extension: 95 °C for 15 seconds > 55 °C for 15 seconds > 72 °C for 10 seconds (28 cycles). Splicing analysis in hMNPs was performed as described above. After the PCR, 3.5 µL of 100 bp DNA Ladder (Promega, #G210A) plus 1x Blue/Orange Loading Dye (Promega, #G190A) and 8 µL of each PCR reaction plus 1x Blue/Orange Loading Dye were loaded on a 2% agarose gel run at 150 V. Gel was developed using the GelDoc Go Imaging System (Biorad). Splicing analysis of CHK1 mRNA in FUS mouse spinal cords and mMNs was performed via semi-quantitative PCR. PCR samples were set up as follows: 20 ng cDNA template derived from mouse spinal cord or mMNs (see ‘RNA extraction and cDNA synthesis in mice and mMNs’ section), 5 µL 5x MyTaq Reaction Buffer (Bioline, BIO-37112), 0.5 µL MyTaq HS DNA Polymerase (Bioline, BIO-21111) and 4 µL of gene-specific forward and reverse primers (5 µM each) in a final volume of 25 µL. The PCR cycling conditions were the following: initial denaturation: 95 °C for 2 minutes (one cycle), denaturation: 95 °C for 15 seconds, annealing: 15 seconds at 57 °C for CHK1 (exons 2-4) and 55 °C for GAPDH, extension: 72 °C for 10 seconds. For CHK1, 5 µL of PCR reaction were collected after 25, 28, and 31/32 cycles of denaturation/annealing/extension while for GAPDH, 5 µL were collected after 20 and 24 (FUS mouse) or 20 and 22 (mMNs) cycles. Each sample was loaded on a 2% agarose gel. For a complete list of primers used in this study see Table 2.

### Mice

Mice were housed at the Tor Vergata University Animal Facility (CIMETA) in accordance with the FELASA Recommendations, European Guidelines for the use of animals in research (2010/63/EU), and Italian Laws (D.L. 26/2014). They were kept at a constant temperature of 22 ± 1 °C, relative humidity of 50%, and a 12 h light cycle (7 a.m.–7 p.m.), with free access to food and water. To ensure nutrition and hydration, wet food was provided to cages where mice displayed signs of paralysis. Hemizygous Tg (Prnp-FUS) WT3Cshw/J mice expressing human wild-type FUS (+/− hFUS, Jackson Laboratories), displaying no pathological signs, were backcrossed to obtain homozygous mice (+/+ hFUS), which show ALS phenotypes and early death at 38–40 days of age. Genotyping was performed as previously described [39]. All experiments were conducted in compliance with the ARRIVE guidelines, the European Guidelines (2010/63/EU) and the requirements of Italian laws (D.L. 26/2014), under the approval by the Italian Ministry of Health (protocol number 383/2022 PR). Every effort was made to minimize mice suffering and reduce the number of mice used to obtain reliable results. A total of *n* = 6 mice were included in the study.

### Data visualization and statistical analyses

GraphPad Prism v9 was used to generate graphs and perform statistical analysis. Immunofluorescence results are presented as superplots [40]. Every grey dot of the dot plot represents one cell nucleus. The different shades of grey represent each independent experiment. Outliers were occasionally removed using the Prism ROUT function with Q = 1%. Next, the average of the number of foci per nucleus or the relative mean nuclear intensity was calculated for each condition and was plotted. In this dot plot, each red dot represents the mean of the number of foci per nucleus or the mean of the relative mean nuclear intensity for each condition in each experiment, whereas error bars represent the mean ± SEM. Statistical analyses were performed on the mean plots. The two dot plots were then overlayed using the GraphPad Prism Layout function. Ordinary one-way ANOVA was used to calculate p-values. When only two conditions had to be compared, unpaired t test was used to calculate *p*-values.

## Results

### CHK1 and ASF1A proteins are downregulated in cells bearing FUS and TDP-43 CIs

**Fig. 1:**
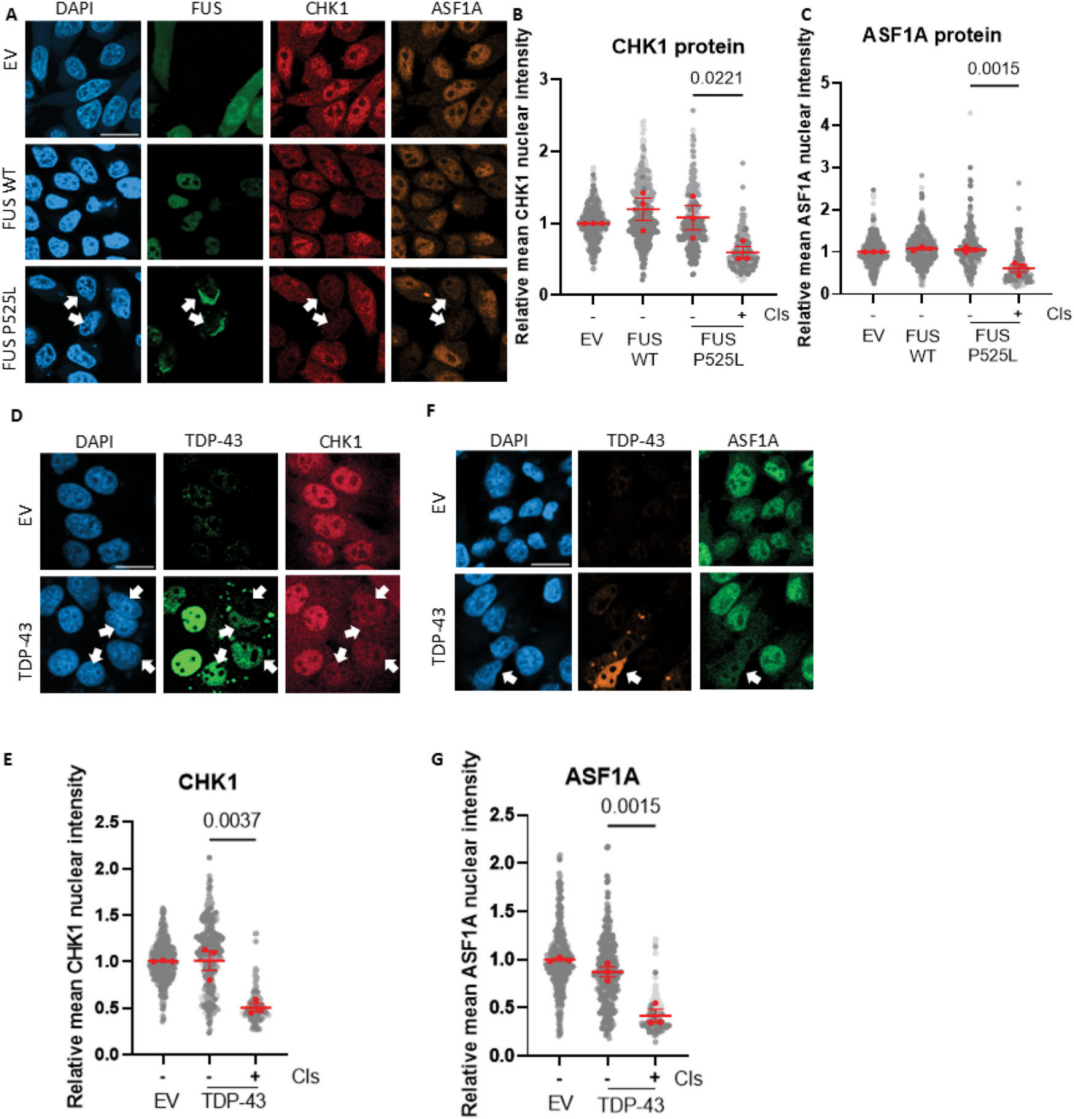
CHK1 and ASF1A proteins are downregulated in cells bearing FUS and TDP-43 CIs. **A** Representative images of HeLa cells overexpressing WT or mutant P525L-FUS or transfected with an EV and probed for FUS, CHK1 and ASF1A. DNA was counterstained with DAPI. White arrows indicate representative cells showing the described phenotype. Scale bar is 20 µm. **B** Quantification of CHK1 protein nuclear intensity in cells treated as in (**A**). Red dots and error bars are mean ± SEM. *N* = three independent experiments. At least 50 cells per condition were analysed. **C** Quantification of ASF1A protein nuclear intensity in cells treated as in A. Red dots and error bars are mean ± SEM. *N* = three independent experiments. At least 50 cells per condition were analysed. **D** Representative images of cells overexpressing WT TDP-43 or transfected with an EV and probed for TDP-43 and CHK1. DNA was counterstained with DAPI. White arrows indicate cells bearing CIs. Scale bar is 20 µm. **E** Quantification of CHK1 protein nuclear intensity in cells treated as in (**D**). Red dots and error bars are mean ± SEM. *N* = three independent experiments. At least 50 cells per condition were analysed. **F** Representative images of cells overexpressing WT TDP-43 or transfected with an EV and probed for TDP-43 and ASF1A. DNA was counterstained with DAPI. White arrows indicate representative cells showing the described phenotype. Scale bar is 20 µm. **G** Quantification of ASF1A protein nuclear intensity in cells treated as in (**F**). Red dots and error bars are mean ± SEM. *N* = three independent experiments. At least 50 cells per condition were analysed.

In our previous work, we found that the formation of FUS and TDP-43 CIs exerts a genotoxic gain of function and leads to accumulation of DSBs by altering DDR signalling [32]. However, the multiple DDR defects associated with the formation of FUS and TDP-43 CIs causing such DNA damage accumulation have not been completely unravelled yet. In this study, we set out to determine additional mechanisms underlying such phenotype. CHK1 is widely known as an important player in the response to replication stress as a target of ATR [24]. Nevertheless, a paper published many years ago showed that chemical inhibition of CHK1 results in accumulation of DNA breaks and increased apoptosis in differentiated rat cortical neurons [41]. Additionally, CHK1 was shown to play an important role in NHEJ in non-replicating cells [26]. Hence, we decided to investigate whether the formation of FUS and TDP-43 CIs might negatively impact CHK1 protein levels. To address this question, we transfected HeLa cells with the FUS split system [32, 35] in which WT or P525L mutant form of FUS are cloned in frame to the N-terminal YFP protein fragment mVenus (mVen) or the CFP C-terminal protein fragment mCerulean (mCer). Upon natural interaction between two FUS proteins, the resulting fluorescence signal emits in the green channel. Fluorescence localizes in the nucleus in cells overexpressing WT FUS while highly fluorescent CIs form in cells overexpressing the mutant FUS-P525L protein [32]. Transient overexpression of both WT and mutant P525L-FUS [8], results a in a 4 or 5 fold increase in total FUS levels (Fig. S1A-C with the uncropped western blot as supplementary material). We found that approximately 40% of cells overexpressing mutant P525L-FUS generate CIs while cells overexpressing WT FUS or transfected with an EV do not (Fig. S1D). This indicates that CIs only form upon overexpression of mutant FUS, whereas transfection per se nor transient overexpression of WT FUS is sufficient to induce CIs in cell lines. Importantly, induction of exogenous DNA damage by treatment with the radiomimetic drug neocarzinostatin (NCS) does not increase the frequency of CI formation (Fig. S1D), suggesting that CIs tend to form prior and independently from DNA damage accumulation. Consistent with our previous findings [32], cells bearing mutant FUS CIs exhibit a diffuse and intense pan-nuclear γH2AX signal and a complete loss of 53BP1 foci upon NCS treatment, while the surrounding cells equally transfected but lacking CIs form decrete 53BP1 foci (Fig. S1E, F). We next wondered whether CHK1 and ASF1A protein levels were affected in CI positive cells. Therefore, we performed indirect immunofluorescence (IF) experiments in cells transfected with an EV or WT or mutant P525L-FUS probing for total CHK1 and ASF1A proteins. Intriguingly, we found a reduced nuclear levels of both proteins in cells bearing CIs but not in the other cells of the same sample(Fig. 1A-C). Hence, we hypothesized that loss of a consistent fraction of CHK1 protein could be one cause of DNA damage accumulation in cells experiencing CIs. We then investigated whether the reduced nuclear levels of CHK1 and ASF1A proteins were due to lower transcript levels. To test this, we first sorted fluorescent cells transfected with plasmids expressing WT FUS or P525L-FUS split system to isolate transfected cells overexpressing WT FUS or enrich for cells bearing fluorescent CIs induced by expression of mutant FUS. As expected, the WT FUS split system results in a diffused nuclear GFP signal, while expression of P525L FUS fused with mVen or mCer gives rise to fluorescent GFP puncta in the cytoplasm of cells. Cell sorting was followed by RT-qPCR analyses to determine *CHK1* and *ASF1A* mRNA levels in cells overexpressing either WT (no CIs) or P525L FUS (with CIs). Importantly, we found that neither *CHK1* nor *ASF1A* transcript levels were reduced in the presence of CIs (Fig. S1G, H). It has been shown that CHK1 protein stability is reduced upon an exon 3 skipping, alternative splicing (AS) event. This event is regulated by TRA2A/B proteins [42], which have been identified as FUS interactors [43]. Therefore, we wondered if CHK1 exon 3 skipping may be induced by the expression of mutant FUS and the formation of FUS CIs. To test this hypothesis, we performed RT-PCR with primers flanking exon 3 in *CHK1* mRNA using RNA extracted from GFP-sorted cells. Following gel electrophoresis, we observed only one PCR product running as a band of 327 bp, corresponding to the *CHK1* isoform in which exon 3 is included, across all conditions tested (Fig. S1I with the uncropped agarose gel available as supplementary material). No shorter bands appeared in cells expressing P525L FUS, indicating that *CHK1* mRNA does not undergo AS in these cells. Differently, knockdown of TRA2A/B resulted in exone 3 skipping giving a 107bp PCR product as previously observed in [42], thus providing a positive control in our assay (Fig. S1I). Given that the vast majority of sALS patients exhibit CIs composed of TDP-43 [3] and that the formation of TDP-43 CIs is associated with a similar genotoxic gain-of-function [32], we investigated whether nuclear levels of CHK1 and ASF1A proteins were altered also in cells harbouring TDP-43 CIs. By performing IF in cells transfected with WT TDP-43, we found that also TDP-43 CIs correlate with a reduction of CHK1 and ASF1A nuclear protein levels (Fig. 1D-G). Moreover, we investigated if overexpression of TDP-43 carrying ALS-associated mutations also impinges on CHK1 nuclear levels. In HeLa cells, we achieved approximately 4 to 6-fold overexpression levels of either WT or three different ALS-associated mutant versions of TDP-43 (A382T TDP-43, I383V TDP-43, and M337V TDP-43) (Fig. S1K–M with the uncropped blot available as additional supplementary material) and we found that the formation of CIs by TDP-43 mutants also led to CHK1 downregulation (Fig. S1N). Quantification of CHK1 intensity revealed that CIs generated by both WT and mutant TDP-43 induce CHK1 downregulation to a comparable extent (Fig. S10). In addition, we monitored the effects of TDP-43 mutants overexpression on genome integrity by staining for the DNA damage marker γH2AX. Similarly to CHK1 levels, we found that cells bearing TDP-43 mutant CIs show increased γH2AX nuclear intensity to a comparable extent respect to cells bearing WT TDP-43 CIs (Fig. S1N, P). Only cells bearing CIs show CHK1 downregulation while cells overexpressing the mutant alleles but lacking CIs fail to downregulate CHK1 or accumulate DNA damage, similarly to what we observed for WT TDP-43 (Fig. S1N–P). These results are in agreement with previous findings from our groupsuggesting that TDP-43 mutation does not exacerbate genotoxic effects of CI nor co-localization between TDP-43 CI and stress granule markers [32].

Taken together, these results show that formation of FUS and TDP-43 CIs results in decreased CHK1 and ASF1A proteins stability without impacting on their transcription. Additionally, CHK1 downregulation in cells bearing FUS CIs is not caused by alterations in the AS of its transcript and consequent protein’s stability deregulation. Finally, we observed that overexpression of WT and ALS-linked mutated TDP-43 leads to a comparable decrease in CHK1 levels and similar γH2AX accumulation. Given that WT TDP-43 form CIs in the majority of sporadic ALS cases and that overexpression of TDP-43 mutants produce a similar phenotype, as also demonstrated by our previous work [32], we decided to focus on WT TDP-43 only for the remainder of the study.

### CHK1 and ASF1A are downregulated in neuronal cell lines and in a FUS-ALS mouse model

**Fig. 2:**
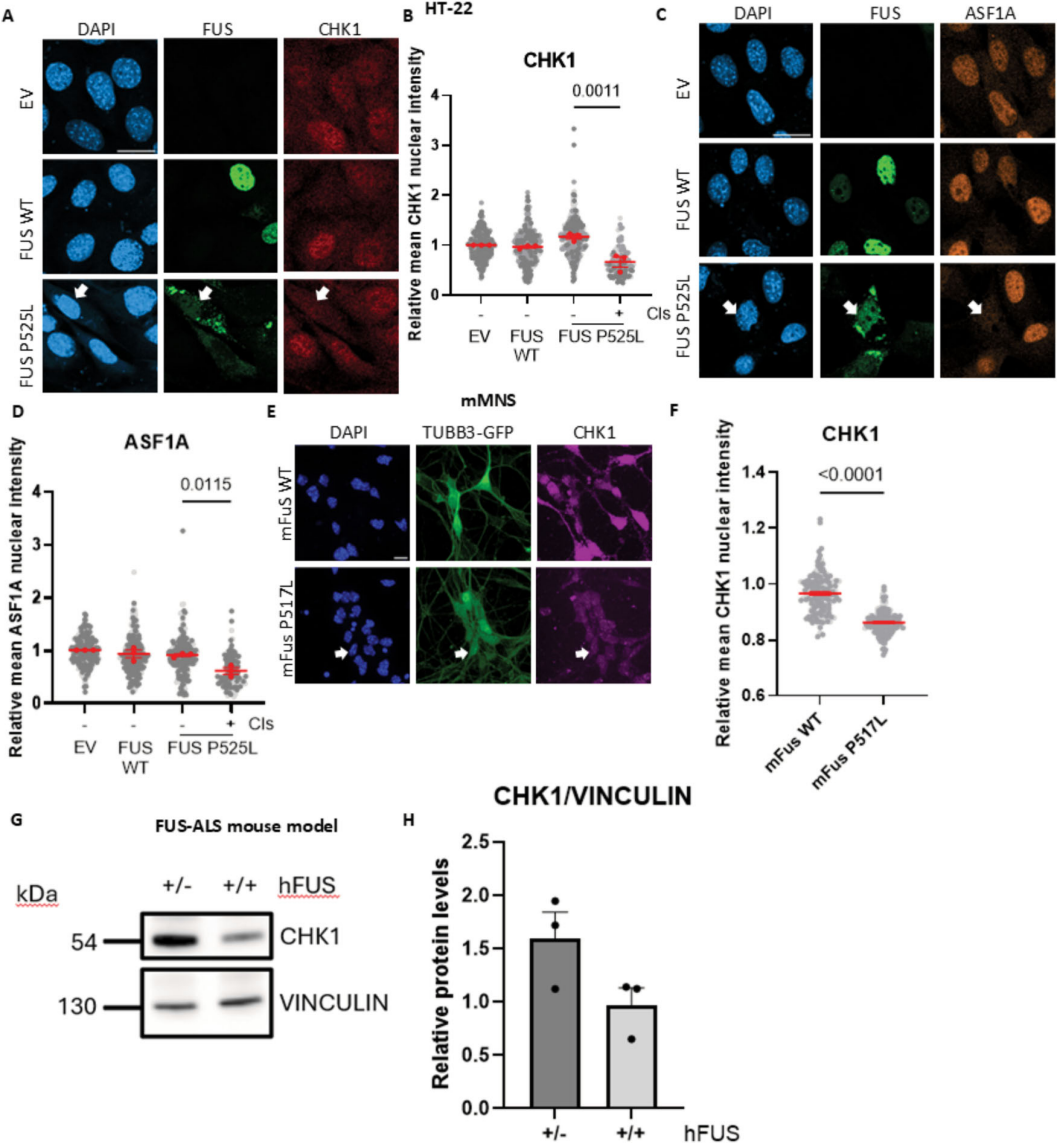
CHK1 and ASF1A proteins are downregulated in neuronal cells bearing FUS CIs and in a mouse model of FUS-ALS. **A** Representative images of HT-22 cells overexpressing either WT or mutant P525L FUS or transfected with an EV and probed for FUS and Chk1. DNA was counterstained with DAPI. White arrows indicate representative cells showing the described phenotype. Scale bar is 20 µm. **B** Quantification of Chk1 protein nuclear intensity in cells treated as in (**A**). Red dots and error bars are mean ± SEM. *N*= three independent experiments. At least 50 cells per condition were analysed. **C** Representative images of HT-22 cells treated as in (**A**) and probed for FUS and Asf1A. DNA was counterstained with DAPI. White arrows indicate representative cells showing the described phenotype. Scale bar is 20 µm. **D** Quantification of Asf1A protein nuclear intensity in cells treated as in (**C**). Red dots and error bars are mean ± SEM. *N*= three independent experiments. At least 50 cells per condition were analysed. **E** Representative images of mature murine motor neurons (mMNs) carrying either WT (mFus WT) or mutant (mFus P517L) murine *Fus* gene and probed for ubb3 and Chk1. DNA was counterstained with DAPI. White arrows indicate representative cells showing the described phenotype. Scale bar is 100 µm. **F** Quantification of Chk1 nuclear intensity in cells treated as in (**E**). Red bars are mean ± SEM. *N*= two independent experiments. At least 130 cells per condition were analysed. **G** Representative western blot showing Chk1 protein levels in spinal cord samples derived from mice expressing human WT FUS (hFUS) in either heterozygosity (+/- hFUS) or homozygosity (+/+ hFUS). Vinculin was used as loading control. **H** Quantification of (**G)**. Error bars are mean ± SEM. *N*= three mice per group.

**Fig. 3:**
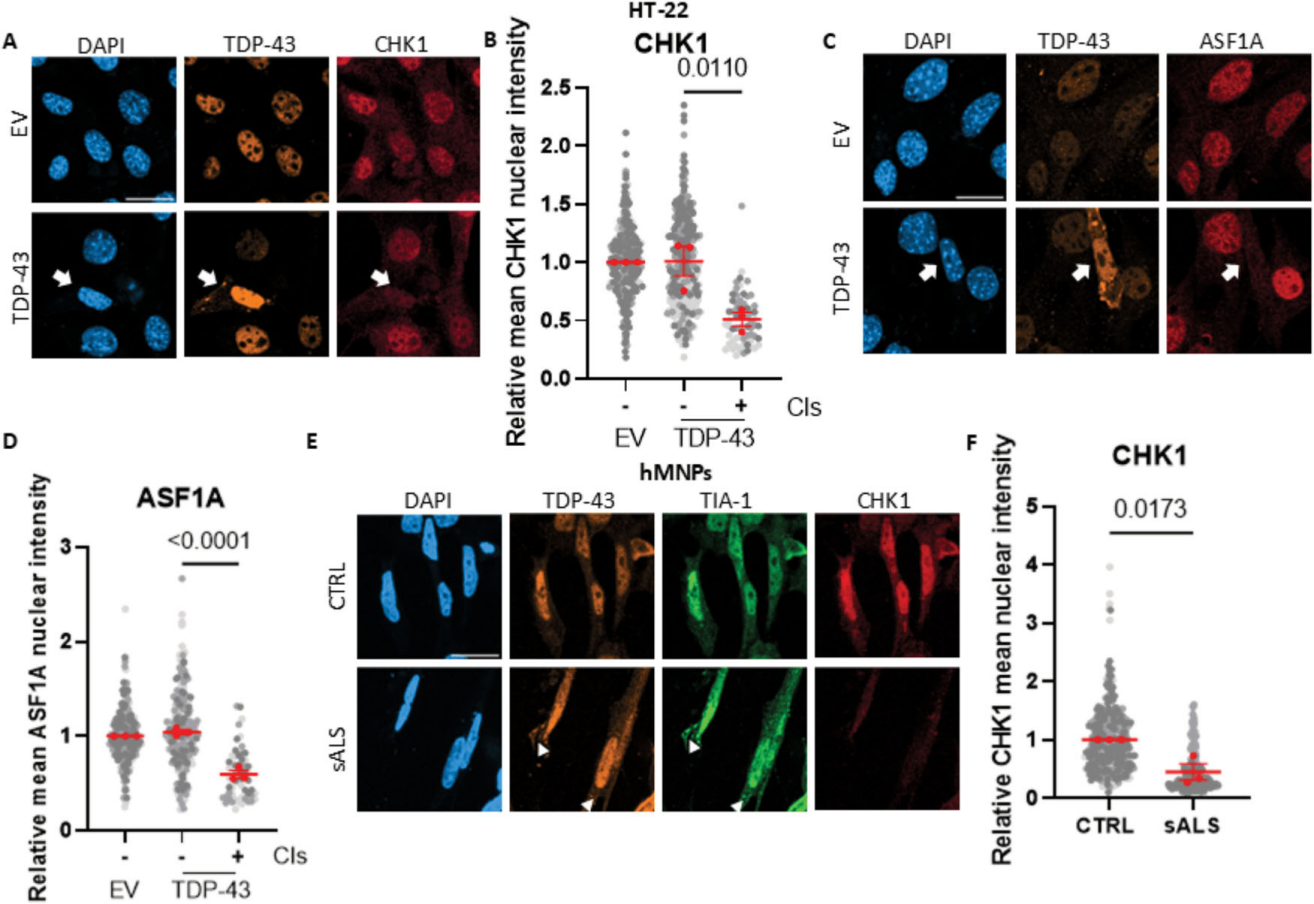
CHK1 and ASF1A proteins are downregulated in neuronal cells bearing TDP-43 CIs and in sporadic patient-derived MNPs. **A** Representative images of HT-22 cells overexpressing TDP-43 or transfected with an EV and probed for TDP-43 and Chk1. DNA was counterstained with DAPI. White arrows indicate representative cells showing the described phenotype. Scale bar is 20 µm. **B** Quantification of Chk1 nuclear intensity in cells treated as in (**A**). Red dots and error bars are mean ± SEM. *N*= three independent experiments. At least 50 cells per condition were analysed. **C** Representative images of HT-22 cells treated as in (**A**) and probed for TDP-43 and Asf1A. DNA was counterstained with DAPI. White arrows indicate representative cells showing the described phenotype. Scale bar is 20 µm. **D** Quantification of Asf1A nuclear intensity in cells treated as in (**C**). Red dots and error bars are mean ± SEM. *N*= three independent experiments. At least 50 cells per condition were analysed. **E** Representative images of hMNPs derived from a healthy control (CTRL) or from a sporadic ALS patient (sALS) and probed for TDP-43, TIA-1 and CHK1. DNA was counterstained with DAPI. White arrowheads indicate co-localisation between TDP-43 and TIA-1 cytoplasmic puncta. Scale bar is 20 µm. **F** Quantification of CHK1 protein nuclear intensity in cells treated as in (**E**). Red dots and error bars are mean ± SEM. *N*= three independent experiments. At least 30 cells per condition were analysed.

Building on the previous results obtained, we aimed to test whether these findings are also relevant for neuronal cells. To this end, we analyzed CHK1 and ASF1A protein levels in the murine, primary hippocampal neuronal cell line HT-22 experiencing FUS CIs. In these cells, we achieved transfection efficiency and frequency of CIs induction comparable to HeLa cells (Fig. S2A–C with the uncropped blot available as additional supplementary material). Consistently with our previous observations, we found that in HT-22 cells, the formation of FUS CIs also led to reduced nuclear levels of Chk1 and Asf1A (Fig. 2A–D), along with a roughly three-fold increase in γH2AX intensity (Fig. S2D) and impaired 53BP1 foci formation (Fig. S2E, F). We then decided to investigate CHK1 nuclear levels in other more robust ALS-linked cellular model systems. We started by performing IF analyses in mature murine motor neurons (mMNs) derived from mouse embryonic stem cells (mESCs) expressing either the WT form (mFus WT) or the mutant P517L Fus (mFus P517L, equivalent to the human P525L FUS mutation). In our previous work, we found that these cells accumulate γH2AX and show a defective phospho-53BP1 foci formation [32]. Consistently, we found reduced nuclear levels of Chk1 also in the Fus P517L mMNs cells (Fig. S2E, F). We also tested if C1 protein downregulation was caused by decreased levels of its transcript or due to the exclusion of Exon 3 in the mature mRNA. Similarly to HeLa cells, the level of *Chk1* mRNA was not affected (Fig. S2G) and no AS with skipping of the Exon 3 was observed in mutant mFus P517L mMN (Fig. S2H and the uncropped gel available as additional supplementary material). Therefore, we conclude that CHK1 downregulation is associated with FUS ALS-link mutation or dysfunctions in all three cell types tested, including mMNs. In previous studies we found an increased percentage of γH2AX-positive and less 53BP1-positive nuclei also in tissue samples from the spinal cord of a murine model of FUS-linked ALS [32]. Therefore, we wondered if Chk1 loss could also explain DNA damage accumulation in this preclinical model system. To test this hypothesis, we performed western blotting analyses in samples derived from the lumbar spinal cord of heterozygous non-symptomatic and homozygous symptomatic hFUS animals [39]. Excitingly, we found that samples derived from homozygous, symptomatic animals display reduced levels of Chk1 in their spinal cord (Fig. S2G, H and the uncropped gel available as additional supplementary material). Furthermore, consistently with all the cellular systems tested, we did not observe decreased *Chk1* transcript levels (Fig. S2I) nor altered AS (Fig. S2J, K and the uncropped gels available as supplementary material) in samples from symptomatic animals of this preclinical mouse model. We then wondered if also formation of TDP-43 CIs could affect CHK1 nuclear levels in neuronal cell lines. To address this question, we overexpressed TDP-43 in HT-22 cells and performed IF to probe for Chk1 and Asf1A. Consistently with our previous results, we found that formation of TDP-43 CIs leads to reduced Chk1 and Asf1A protein levels also in HT-22 neuronal cells (Fig. 3A–D). Finally, we investigated whether CHK1 was downregulated in a sporadic ALS (sALS) cellular model system. To this end, we investigated CHK1 nuclear levels in human motor neurons progenitors (hMNPs) derived from a sALS patient (or an age- and sex-matched control [33]) in which we previously observed TDP-43 cytoplasmic mislocalization and accumulation of DNA damage [32]. By IF, we found that TDP-43 is de-localised in the cytoplasm in sALS cells, with some of them showing small TDP-43 cytoplasmic puncta co-localising with the stress granule marker TIA-1 (white arrowheads in Fig. 3E), as previously reported [32, 44]. Intriguingly, when we probed for CHK1 protein level, we found that CHK1 is strongly reduced in the nuclei of cells derived from the sALS patient with TDP-43 cytoplasmic mislocalization if compared to control cells (Fig. 3E, F). We also investigated *CHK1* mRNA levels in this cellular model system and found no downregulation of *CHK1* transcript (Fig. S3A). With regards to AS of *CHK1* exon 3 in hMNPs, we observed a major 327 bp-long PCR product and an extremely faint band of approximately 100 bp (Fig. S3B with the uncropped gel available as additional supplementary material), which corresponds to the *CHK1* isoform in which exon 3 is skipped [42]. We thus proceeded to quantify the percentage of splicing inclusion (PSI) to determine whether CHK1 protein downregulation might be caused by alteration in its AS. However, we found no significant differences in the relative fraction of CHK1 exon 3 inclusion in sALS samples compared to control hMNPs (Fig. S3C).

In conclusion, we observed CHK1 and ASF1A downregulation in several cellular model systems of FUS- and TDP-43-linked ALS pathology and in a in vivo FUS-ALS preclinical mouse model. In all systems tested, CHK1 protein downregulation is not caused by reduced levels of its transcript nor by aberrant AS of exon 3. Importantly, the observation that CHK1 downregulation also occurs in hMNPs suggests that CHK1 loss and the consequent loss of genome integrity, may be an early event in ALS pathogenesis, consistent with previous findings [20].

### CHK1 loss has a causal role in DNA damage accumulation

**Fig. 4:**
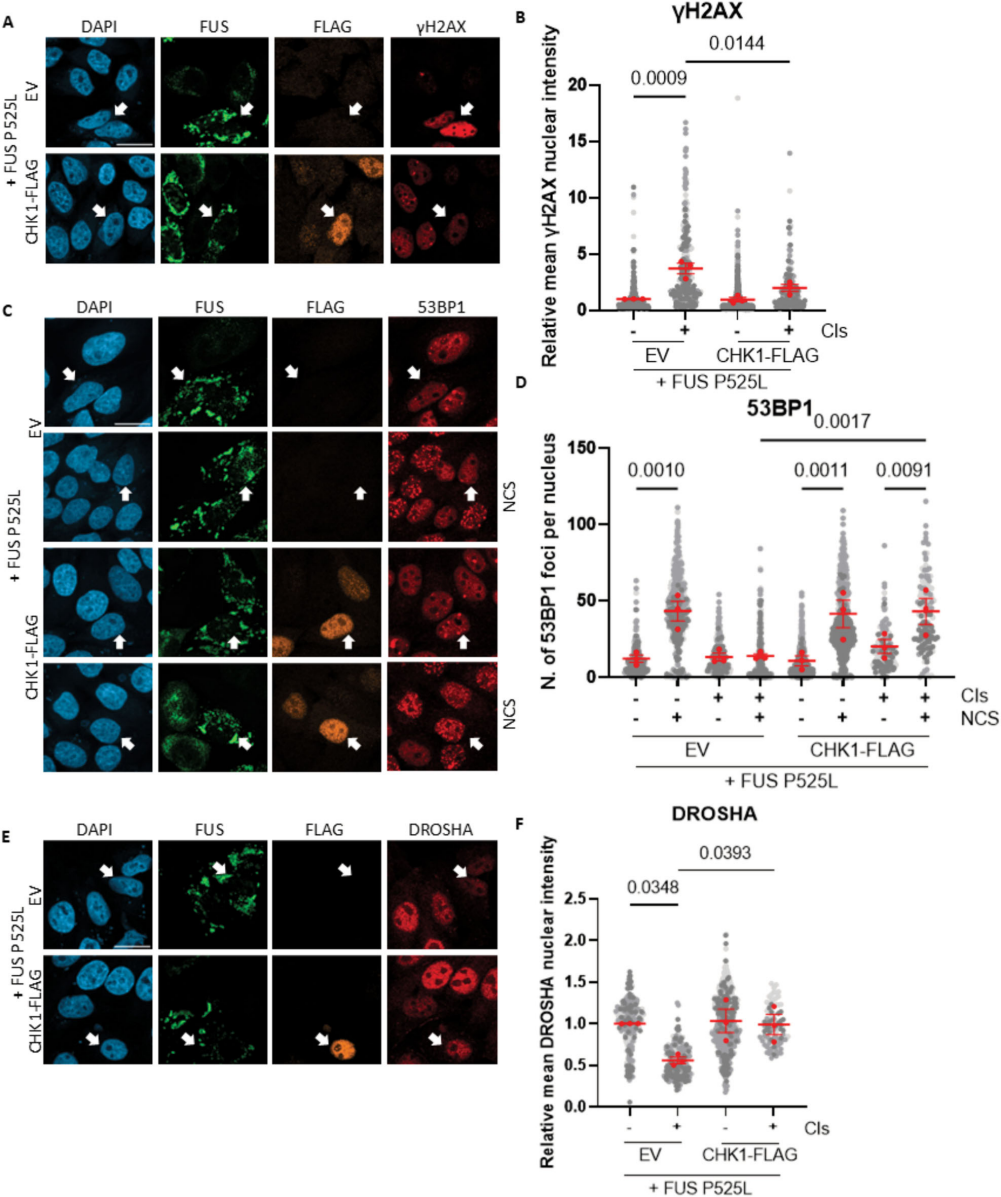
CHK1 overexpression reduces γH2AX accumulation and restores 53BP1 foci formation and DROSHA level in cells bearing FUS CIs. **A** Representative images of HeLa cells overexpressing mutant P525L FUS together with a plasmid encoding for FLAG-tagged CHK1 (CHK1-FLAG) or an EV and probed for FUS, FLAG and γH2AX. DNA was counterstained with DAPI. White arrows indicate representative cells showing the described phenotype. Scale bar is 20 µm. **B** Quantification of γH2AX nuclear intensity in cells treated as in (**A**). Red dots and error bars are mean ± SEM. *N*= three independent experiments. At least 30 cells per condition were analysed. **C** Representative images of HeLa cells overexpressing mutant P525L FUS together with a plasmid encoding for FLAG-tagged CHK1 or an EV, treated or not with NCS and probed for FUS, FLAG and 53BP1. DNA was counterstained with DAPI. White arrows indicate representative cells exhibiting the described phenotype. Scale bar is 20 µm. **D** Quantification of the number of 53BP1 foci per nucleus in cells treated as in (**C**). Red dots and error bars are mean ± SEM. *N*= three independent experiments. At least 30 cells per condition were analysed. **E** Representative images of HeLa cells treated as in (**A**) and probed for FUS, FLAG and DROSHA. DNA was counterstained with DAPI. White arrows indicate representative cells showing the described phenotype. Scale bar is 20 µm. **F** Quantification of DROSHA protein nuclear intensity in cells treated as in (**E**). Red dots and error bars are mean ± SEM. *N*= three independent experiments. At least 30 cells per condition were analysed.

Given that CHK1 and ASF1A have been shown to play a role in NHEJ [26] we hypothesized that the down regulation of CHK1 and ASF1A could contribute to the accumulation of DNA damage in cells bearing CIs. To test this hypothesis, we restored CHK1 and ASF1A nuclear levels by transiently over-expressing them in HeLa cells expressing P525L FUS. By restoring CHK1 nuclear levels, confirmed by both IF and western blotting (Fig. 4A and S4A) (the original uncropped western blot is available as supplementary material), we could reduce γH2AX nuclear signal in cells bearing mutant FUS CIs(Fig. 4A, B). We reasoned that the reduction of γH2AX intensity might be due to a rescue in 53BP1 foci formation. By probing cells for 53BP1 by IF, we found that transient overexpression of CHK1 is sufficient to restore 53BP1 foci formation in damaged cells, despite the persistence of FUS CIs (Fig. 4C, D). Our group identified a role for DROSHA as an important player in the activation of the DDR [15, 17]. Intriguingly, we found that DROSHA protein levels are also reduced in cells bearing FUS CIs [32]. We thus wondered whether DNA damage accumulation due to CHK1 loss may also correlate with reduced DROSHA protein levels and if rescue of CHK1 by overexpression could also restore DROSHA in cells with FUS CIs. Unexpectedly, by IF, we found that CHK1 overexpression in cells bearing FUS CIs also restores DROSHA nuclear levels indicating a possible mechanism for 53BP1 foci rescue (Fig. 4E, F). Since we observed that CHK1 overexpression alone can counteract DNA damage accumulation, we wondered if its depletion via RNA interference could further increase DNA damage signal in cells with CIs. Further supporting a protective role for CHK1 in maintaining genome integrity, depletion of CHK1 by siRNA in cells bearing FUS CIs significantly exacerbates the accumulation of DNA damage in these cells, as indicated by the further increase in γH2AX intensity (Fig. S4B–D). Finally, given that CHK1 promotes NHEJ through ASF1A [26], we wondered if we could reproduce these results by transiently overexpressing ASF1A. To this end, we performed IF and probed cells for γH2AX, 53BP1 and DROSHA in cells overexpressing ASF1A (Fig. S4E and uncropped western blot available as supplementary material). Excitingly, similarly to CHK1, restoration of ASF1A nuclear levels is sufficient to abrogate γH2AX accumulation (Fig. S4F, G) and rescue 53BP1 foci in damaged cells (+NCS) bearing CIs (Fig. S4H, I). Surprisingly, ASF1A overexpression has no impact on DROSHA nuclear levels (Fig. S4J), suggesting that CHK1 can support DNA repair also independently of ASF1A. Finally, we wondered whether we could reproduce these results in cells bearing TDP-43 CIs. We first performed IF to probe for γH2AX and 53BP1. Contrary to our expectations, overexpression of neither CHK1 nor ASF1A (Fig. S4K, N and the complete western blots available as supplementary material) in cells bearing TDP-43 CIs is sufficient to reduce DNA damage accumulation nor to restore 53BP1 foci formation upon exogenous DNA damage (Fig. S4L, M, O, P).

In conclusion, this set of results points to a causative role of CHK1 downregulation in the accumulation of DNA damage in cells bearing FUS CIs, with at least a partial contribution to this phenomenon mediated by the loss of ASF1A. In fact, restoring nuclear levels of both CHK1 and ASF1A is sufficient to reduce γH2AX accumulation and to restore 53BP1 foci formation. Additionally, CHK1, but not ASF1A, preserves DROSHA stability, further supporting DNA repair. These effects may collectively contribute to an efficient repair of DSBs, leading to a reduction of DNA damage accumulation in cells bearing FUS CIs, thus possibly promoting cell survival. In contrast, overexpression of CHK1 and ASF1A in cells bearing TDP-43 CIs has no effect on DNA damage accumulation and does not rescue DDR foci formation. These results suggest that loss of CHK1 and ASF1A is not the main event leading to DNA damage accumulation in cells bearing TDP-43 CIs. Therefore, we directed our subsequent investigations toward FUS CIs.

### Macroautophagy and chaperone-mediated autophagy do not mediate CHK1 degradation in cells bearing FUS CIs

Given the causative role of CHK1 downregulation in the accumulation of DNA damage in cells with FUS CIs, we next set out to investigate which protein degradation pathway is responsible for CHK1 and ASF1A degradation in this context. Autophagy is a major protein homeostasis pathway that is altered in ALS [31]. We started by testing the involvement of macroautophagy using bafilomycin A1 (BafA1) that inhibits later stages of autophagosome-lysosome fusions and also lysosomal degradation [45]. In HeLa cells, we confirmed macroautophagy inhibition by probing for p62 in western blotting analyses (Fig. S5A, B and the uncropped western blot available as supplementary material). We also found that BafA1 does not significantly change the percentage of cells bearing FUS CIs in our settings (Fig. S5C). When we tested whether inhibition of macroautophagy could restore CHK1 nuclear levels, we found that BafA1 treatment does not restore CHK1 in CI-positive cells (Fig. S5D, E). Concomitantly, BafA1 treatment does not reduce γH2AX accumulation (Fig. S5D, F). We then wondered whether chaperone-mediated autophagy (CMA) could be responsible for CHK1 degradation. CMA is a peculiar subtype of autophagy that specifically degrades soluble proteins containing a KFERQ or KFERQ-like motif [46]. CMA has emerged as an important pathway responsible for clearance of aberrant proteins with pathogenic roles in neurodegenerative diseases, including ALS-related proteins ubiquilin-2 and TDP-43 [46–49]. Furthermore, CHK1 is a substrate of CMA, especially in the presence of DNA damage [50]. To inhibit CMA in HeLa cells we used the inhibitor of the heat shock protein HSC70, a key factor in CMA required for selective binding of protein with KFERQ motive, the compound VER-15508 [51]. We confirmed CMA inhibition by western blotting against GAPDH, a known target of this protein degradation pathway (Fig. S5G, H and the uncropped western blot available as supplementary material) and found that VER-15508 treatment had no effects on frequency of CI formation (Fig. S5I). We next performed IF to determine CHK1 and γH2AX levels and found that CMA inhibition does not restore CHK1 nuclear levels nor reduces γH2AX accumulation in cells bearing FUS CIs (Fig. S5J–L).

### The ubiquitin-proteasome system (UPS) mediates CHK1 degradation in cells bearing FUS CIs

**Fig. 5:**
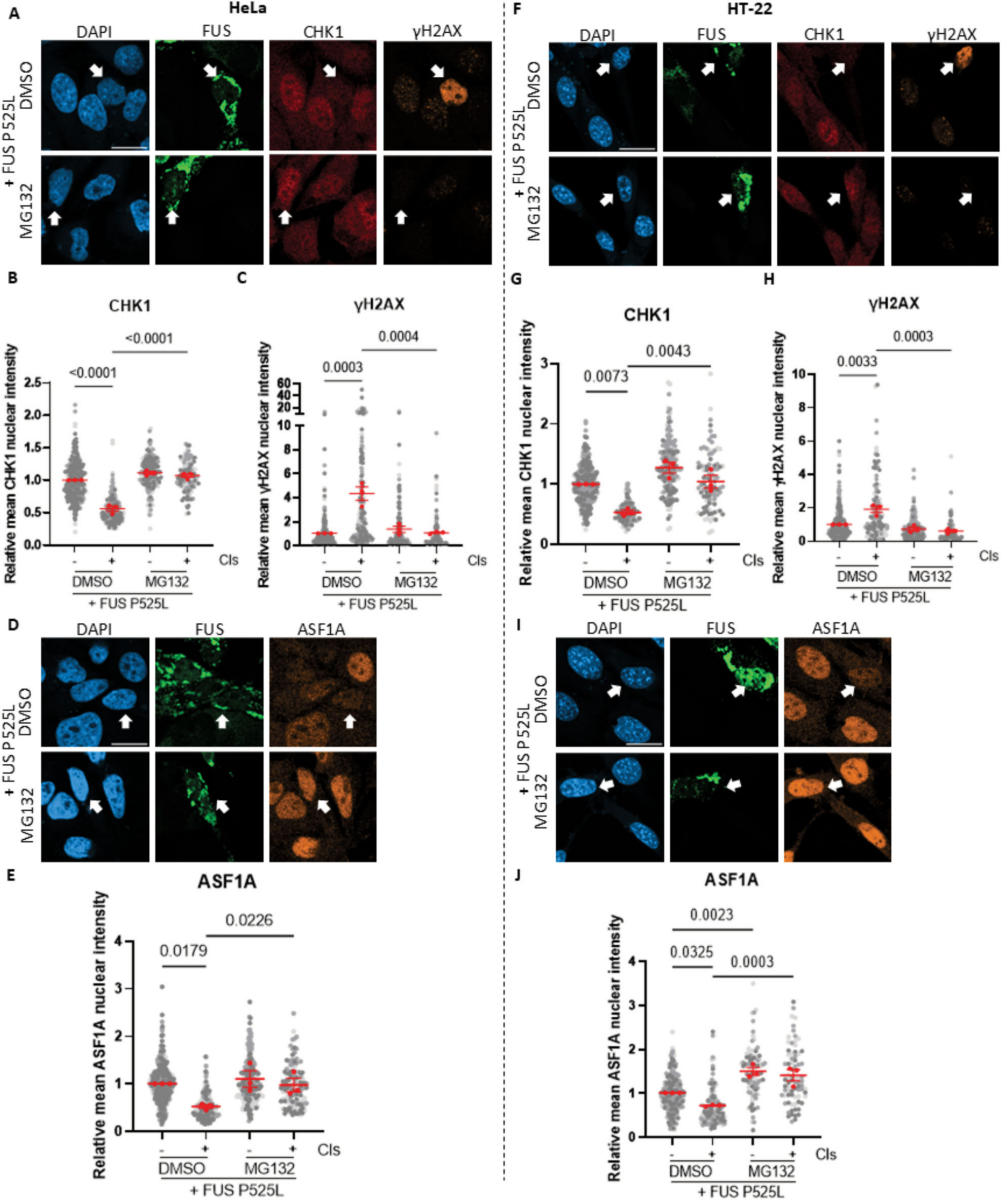
CHK1 and ASF1A are degraded by the proteasome in cells bearing FUS CIs. **A** Representative images of HeLa cells overexpressing mutant P525L FUS and treated with the proteasome inhibitor MG132 or vehicle (DMSO) and probed for FUS, CHK1 and γH2AX. DNA was counterstained with DAPI. White arrows indicate representative cells showing the described phenotype. Scale bar is 20 µm. **B** Quantification of CHK1 nuclear intensity in cells treated as in (**A**). Red dots and error bars are mean ± SEM. *N*= three independent experiments. At least 50 cells per condition were analysed. **C** Quantification of γH2AX nuclear intensity in cells treated as in (**A**). Red dots and error bars are mean ± SEM. *N*= three independent experiments. At least 50 cells per condition were analysed. **D** Representative images of HeLa cells treated as in (**A**) and probed for FUS and ASF1A. DNA was counterstained with DAPI. White arrows indicate representative cells showing the described phenotype. Scale bar is 20 µm. **E** Quantification of ASF1A nuclear intensity in cells treated as in (**D**). Red dots and error bars are mean ± SEM. *N*= three independent experiments. At least 50 cells per condition were analysed. **F** Representative images of HT-22 cells overexpressing mutant P525L FUS and treated with the proteasome inhibitor MG132 or DMSO and probed for FUS, CHK1 and γH2AX. DNA was counterstained with DAPI. White arrows indicate representative cells showing the described phenotype. Scale bar is 20 µm. **G** Quantification of CHK1 nuclear intensity in cells treated as in (**F**). Red dots and error bars are mean ± SEM. *N*= three independent experiments. At least 50 cells per condition were analysed. **H** Quantification of γH2AX nuclear intensity in cells treated as in (**F**). Red dots and error bars are mean ± SEM. *N*= three independent experiments. At least 50 cells per condition were analysed. **I** Representative images of HT-22 cells overexpressing mutant P525L FUS treated as in (**F**) and probed for FUS and ASF1A. DNA was counterstained with DAPI. White arrows indicate representative cells showing the described phenotype. Scale bar is 20 µm. **J** Quantification of ASF1A nuclear intensity in cells treated as in (**I**). Red dots and error bars are mean ± SEM. *N*= three independent experiments. At least 50 cells per condition were analysed.

Given that neither macroautophagy nor CMA seems to be the pathways responsible for CHK1 degradation in our experimental conditions, we next investigated whether the UPS could play this role. To address this question, we used the proteasome inhibitor MG132 in HeLa cells. We confirmed proteasome inhibition by western blotting against ubiquitylated proteins and found that MG132 treatment had no significant effects on CIs formation (Fig. S5M–O and the uncropped western blot available as supplementary material). When we performed IF to investigate CHK1, γH2AX, and ASF1A protein levels in cells treated with vehicle or with MG132, we found that MG132 treatment leads to a complete rescue of CHK1 nuclear levels (Fig. 5A, B) and a marked reduction of γH2AX intensity (Fig. 5A, C) in cells bearing CIs. Moreover, we found that proteasome inhibition also completely restores ASF1A nuclear levels in cells bearing CIs (Fig. 5D, E). MG132 treatment leads to CHK1 accumulation also in untransfected HeLa cells (Fig. S5P, Q), further supporting the notion that CHK1 is indeed degraded via the proteasome, as already reported [52–54]. Given that neuronal cell lines bearing CIs also show CHK1 and ASF1A nuclear loss (Fig. 2), we wondered if the UPS was responsible for CHK1 degradation also in this neuronal context, more relevant for ALS. To this end, we treated HT-22 cells overexpressing P525L FUS with MG132 and performed IF experiments to probe for CHK1 and γH2AX. Excitingly, we found that inhibition of the UPS does not alter CI formation (Fig. S5R–T and the uncropped western blot available as supplementary material) also in HT-22 cells, but is indeed sufficient to rescue CHK1 protein level (Fig. 5F, G) and prevent γH2AX accumulation (Fig. 5F, H). MG132 treatment also restores ASF1A nuclear levels (Fig. 5I, J).

Collectively, this set of results shows that the UPS pathway is responsible for CHK1 and ASF1A degradation in cells experiencing mutant FUS aggregation. Indeed, inhibition of the UPS restores CHK1 and ASF1A nuclear levels and prevents DNA damage accumulation both in non-neuronal and neuronal cell lines. These findings further support the hypothesis that CHK1 loss compromises the maintenance of genome integrity in cells bearing CIs, leading to massive DNA damage signalling, likely contributing to cell death.

## Discussion

Accumulation of DNA damage and defective DNA repair have been linked to neurodegeneration [55]. Accordingly, accumulation of DNA damage and p53 activation is emerging as a novel characteristic of ALS [9, 10]. Consistent with these findings, TDP-43 and FUS have emerged as important players in DNA repair of DNA double strand breaks [21, 22, 56–59], with spinal MNs seeming more sensitive to DNA repair defect associated with to FUS dysfunction, compared to cortical MNs [60]. We also showed accumulation of DNA breaks and aberrant activation of the DDR signalling in different in vitro and in vivo ALS model systems [32]. However, the multiple molecular mechanisms causing accumulation of DNA damage and altered DDR have not yet been elucidated. Many studies have focused on DNA repair defect of loss-of-nuclear function conditions for TDP-43 and FUS [61, 62]. Instead, we tested the hypothesys that the formation of CIs has a unidentified genotoxic gain-of-function, leading to loss of genome integrity aberrant DDR activation possibly leading to death of MNs. by p53 dependent apoptosis Indeed, knockdown of FUS and TDP-43 does not causes evident and rapid accumulation of DNA damage nor alteration in DDR activation as much as accumulation of TDP-43 or mutant FUS CIs. Additionally, in our models, the formation of CIs is not accompanied by loss of nuclear TDP-43 and FUS [32]. These evidences suggest that persistent CIs co-localising with markers of SGs exert a strong genotoxic gain-of-function of CIs with prevails respect to a TDP-43 or FUS loss-of-function. In the present study we investigated molecular causes for the accumulation of DNA damage and aberrant DDR signalling observed in cells bearing FUS and TDP-43 CIs, wich we believe it might represent a usefull proxy of cellular stress experienced by MNs during early stages of fALS and sALS pathology, respectively. We decided to focus on the CHK1-ASF1A axis intrigued by their recently described role in ensuring proper DNA repair of DNA double stranded break [63, 64], also in non-dividing cells [26]. Importantly, CHK1 has been found to sustain survival of differentiated cortical neurons [41]. Consistent with these findings, we found that cells bearing FUS and TDP-43 CIs show reduced nuclear levels of CHK1 and ASF1A proteins, accompanied by accumulation of DNA damage and defective DDR foci formation for the NHEJ mediator factor 53BP1 [32]. The downregulation of nuclear CHK1 and ASF1A at protein levels is not caused by a significant loss of their mRNA transcripts nor by altered regulation of its AS, suggesting that the formation of CIs impact mostly on protein homeostasis. Importantly, we could confirm CHK1 downregulation in different cellular model systems, including the hippocampal neuronal cell line HT-22, mMNs and hMNPs, as well as in samples derived from the spinal cord of a fast progressive FUS-ALS mouse model.

Although cells bearing mutant FUS or TDP-43 CIs exhibit similar phenotypes in terms of DNA damage accumulation and aberrant DDR activation, we found that the role of CHK1 and ASF1A differs in these two context. In fact, upon formation of FUS CIs, both CHK1 and ASF1A downregulation seems to be a key driver of DNA damage accumulation and defective DDR foci formation since the rescue of their level abrogates γH2AX accumulation and restores 53BP1 foci. In contrast, the CHK1-ASF1A axis seems to playa marginal role in the maintenance of genome integrity in cells bearing TDP-43 CIs. In this context, nor overexpression of CHK1 nor ASF1A is sufficient to abrogate γH2AX accumulation or to restore 53BP1 foci formation.This might suggest that the previously identified DROSHA/DDRNAs/53BP1 axis [15, 17] predominantly prevents DNA damage accumulation in cells with TDP-43 pathology. Indeed, TDP-43 and FUS have been identified as DROSHA interactors [65, 66], and we found that cells bearing both FUS and TDP-43 CIs show decreased nuclear levels of DROSHA protein but not of its mRNA [32]. In the present study, we found that CHK1 level restoration is sufficient to reduce DDR signalling and restore the nuclear levels of DROSHA in cells bearing FUS CIs, whereas ASF1A could not. Therefore, CHK1 apparently has multiple roles in DNA repair, not all dependent on ASF1A downstream functions.

Given thepartial requirement of CHK1 and ASF1A in preventing DNA damage accumulation in cells bearing TDP-43 CIs, we decided to focus on mutant FUS CIs to investigate which catabolic pathway was responsible for CHK1 and ASF1A enhanced degradation. By using small molecule inhibitors, we found that the UPS, but not macroautophagy nor CMA, is responsible for CHK1 and ASF1A degradation in cells bearing FUS CIs, consistent with the presence of a cross-talk between the UPS and the need of free ubiquiting in DDR signalling in the context of protein aggregation induced by defective ribosomal products [67]. On top of proteasome-mediated degradation, impaired protein translation also contribute to reducing CHK1 levels in cell bearing CIs. Indeed, we previously showed that FUS CIs co-localise with SGs [32], membrane-less organells sequestering non-translating mRNA and translation factors to protect them during stress [68]. Indeed, ALS-associated FUS mutations (including the P525L mutation) were shown to suppress protein translation and nonsense-mediated RNA decay[69]. Importantly, the restoration of CHK1 and ASF1A nuclear levels through proteasome inhibition completely abolishes accumulation of DNA damage in cells bearing CIs in both neuronal and non-neuronal cells, further confirming a prominent role of CHK1 and ASF1A in maintaining genome integrity upon mutant FUS genotoxic dysfunctions. Importantly, our results are in agreement with previous published literature. Indeed, it was shown that genotoxic stress leads to proteasomal-mediated CHK1 degradation [52], and MG132 was shown to promote neurite extensions in cellular models of mutant TDP-43 pathology [70]. Intriguingly, generation of DSBs was found to promote recruitment of nuclear proteasome activator PA28 at broken DNA ends to favour DNA repair by NHEJ [71]. In light of this finding, we speculate that the formation of FUS CIs leads to accumulation of unrepaired DSBs [32]. In these cells, the generation of DSBs then triggers a deregulated activation of nuclear proteasomes that ultimately leads to CHK1 and ASF1A degradation. Finally, CHK1 and ASF1A loss prevents a proper DDR activation, dampening NHEJ and causing further accumulation of DSBs. It is possible to speculate that accumulation of unrepaired DSBs would feed a vicious cycle, further promoting CHK1 and ASF1A degradation via the proteasome thus exacerbating accumulation of DNA damage. In patients, age or environmental related accumulation of an initial amount of DNA damage could be the trigger of this vicious cycle in MNs, ultimately leading to cell death and contributing to neurodegeneration. In conclusion, our results suggest that restoring CHK1 nuclear levels in MNs of patients with FUS pathology may represent a promising therapeutic avenue to reduce the genotoxic effects caused by the formation of FUS CIs. One possibility is to treat the central nervous system of these patients with small molecules to inhibit the UPS. The use of proteasome inhibitors is already a reality in the clinic for the treatment of other diseases. Indeed, several compounds have been approved for the treatment of multiple myelomas and mantle cell lymphoma, whereas other drugs are being developed for autoimmune diseases [72]. Other than haematological tumours, the use of proteasome inhibitors is now being tested also in brain tumours thanks to the development of new molecules that are able to cross the blood-brain barrier [73].

## Supplementary information

Figure S1 (part 1)

Figure S1 (part 2)

Figure S2 (part 1)

Figure S2 (part 2)

Figure S3

Figure S4 (part 1)

Figure S4 (part 2)

Figure S5 (part 1)

Figure S5 (part 2)

Original uncropped western blots and agarose gels

Supplementary figures legends

Source data

## Data Availability

Uncropped original western blots and agarose gels as well as source data are available as supplementary materials of this article.
