# Supplementary figures and images for: Proteasomal-dependent CHK1 degradation leads to DNA damage accumulation in ALS cellular model systems
Source: Cell Death Dis. 2026 May 6;17(1):599. doi: 10.1038/s41419-026-08603-6 (PMC13315745; doi:10.1038/s41419-026-08603-6)

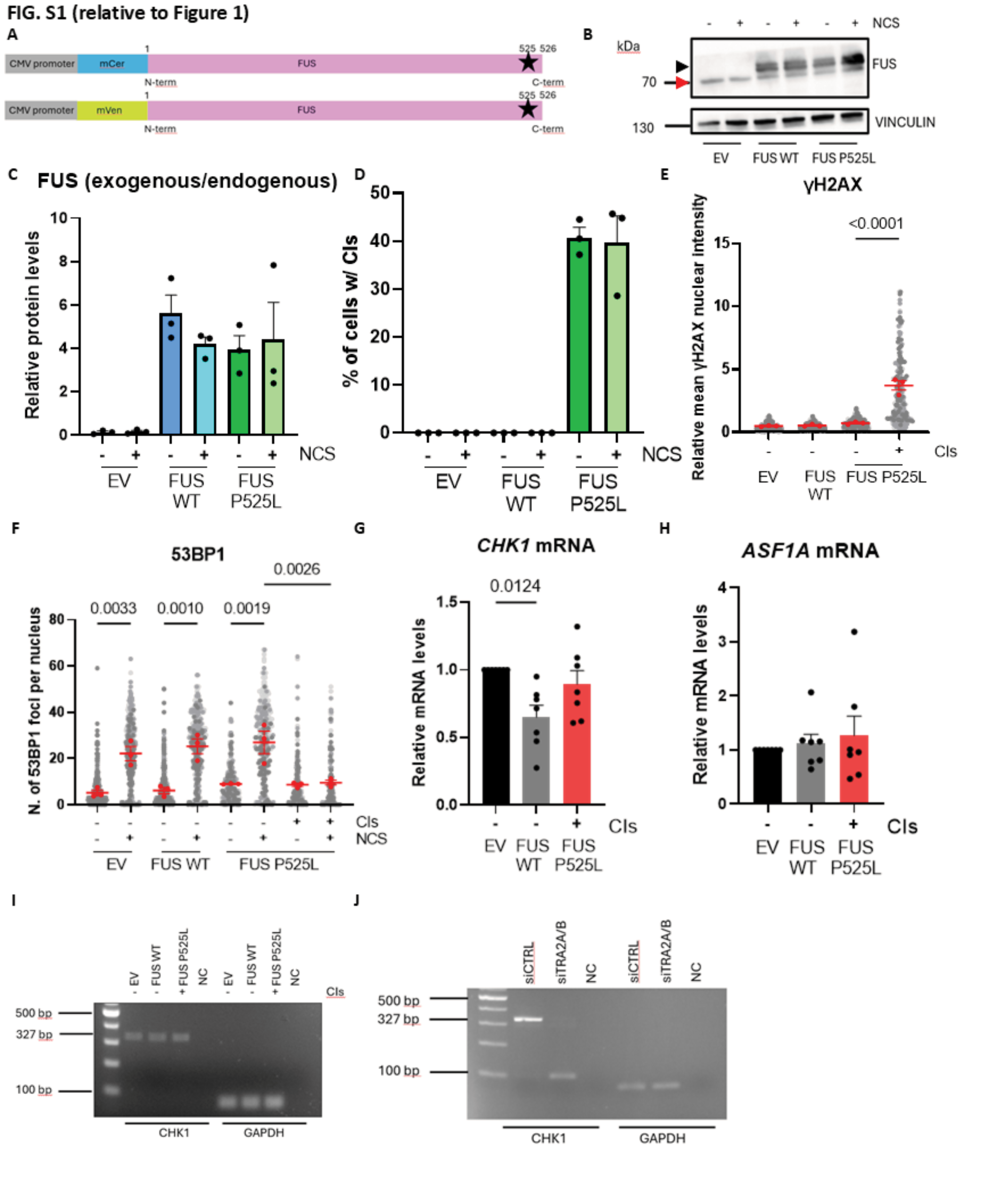

Supplement: Supplementary file 1 — Figure S1 (part 1) [file 41419_2026_8603_MOESM1_ESM.tiff]

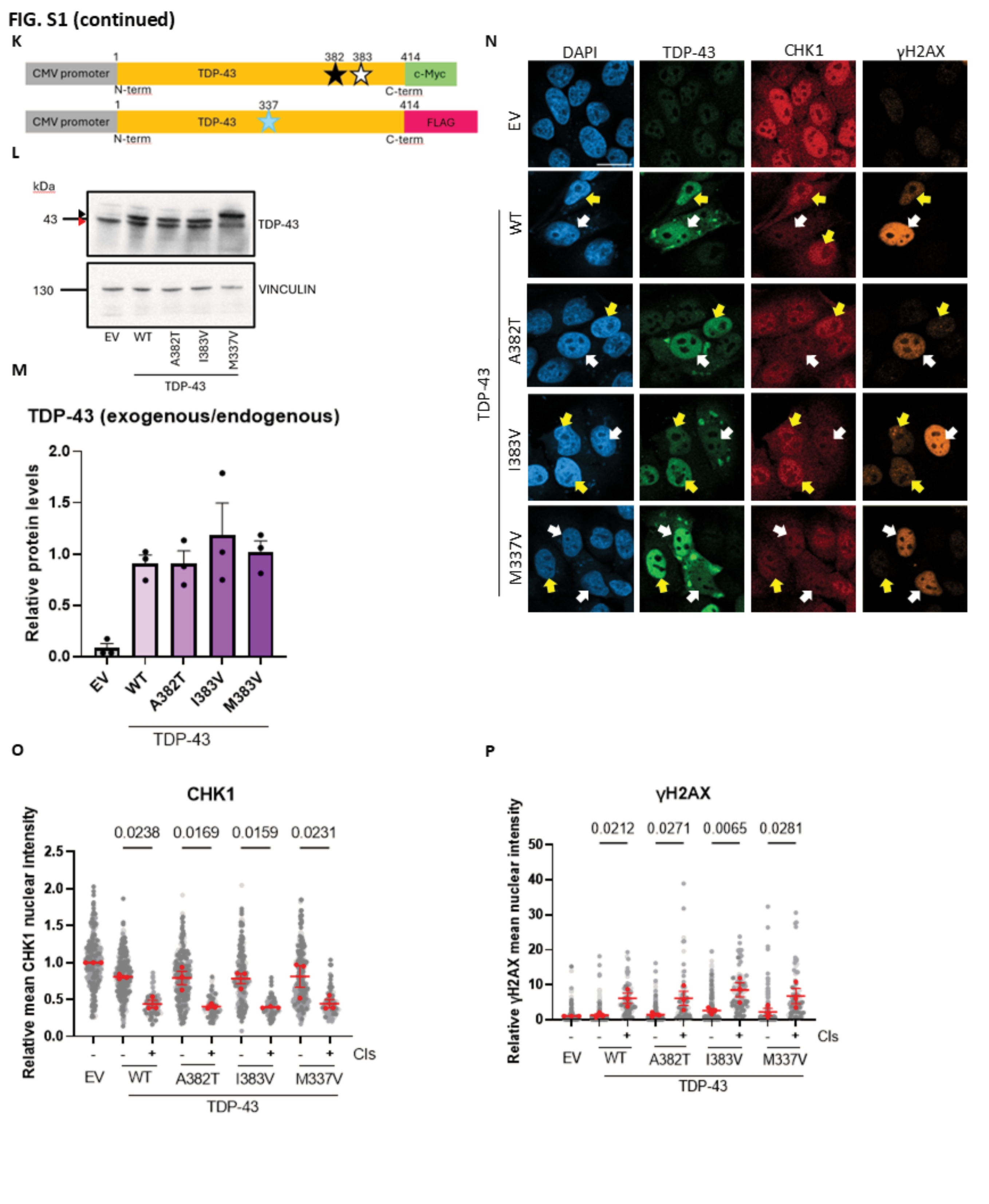

Supplement: Supplementary file 2 — Figure S1 (part 2) [file 41419_2026_8603_MOESM2_ESM.tiff]

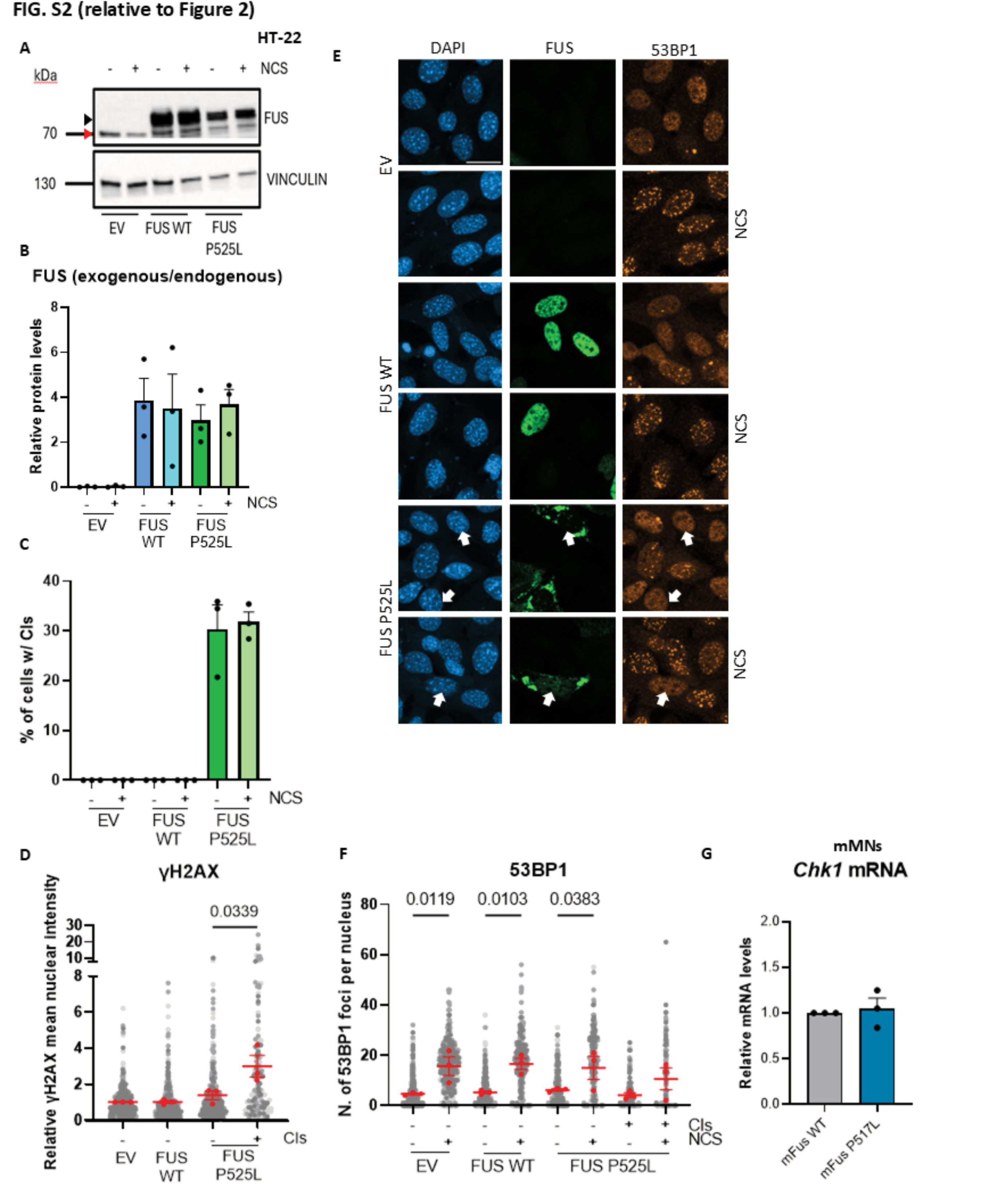

Supplement: Supplementary file 3 — Figure S2 (part 1) [file 41419_2026_8603_MOESM3_ESM.tif]

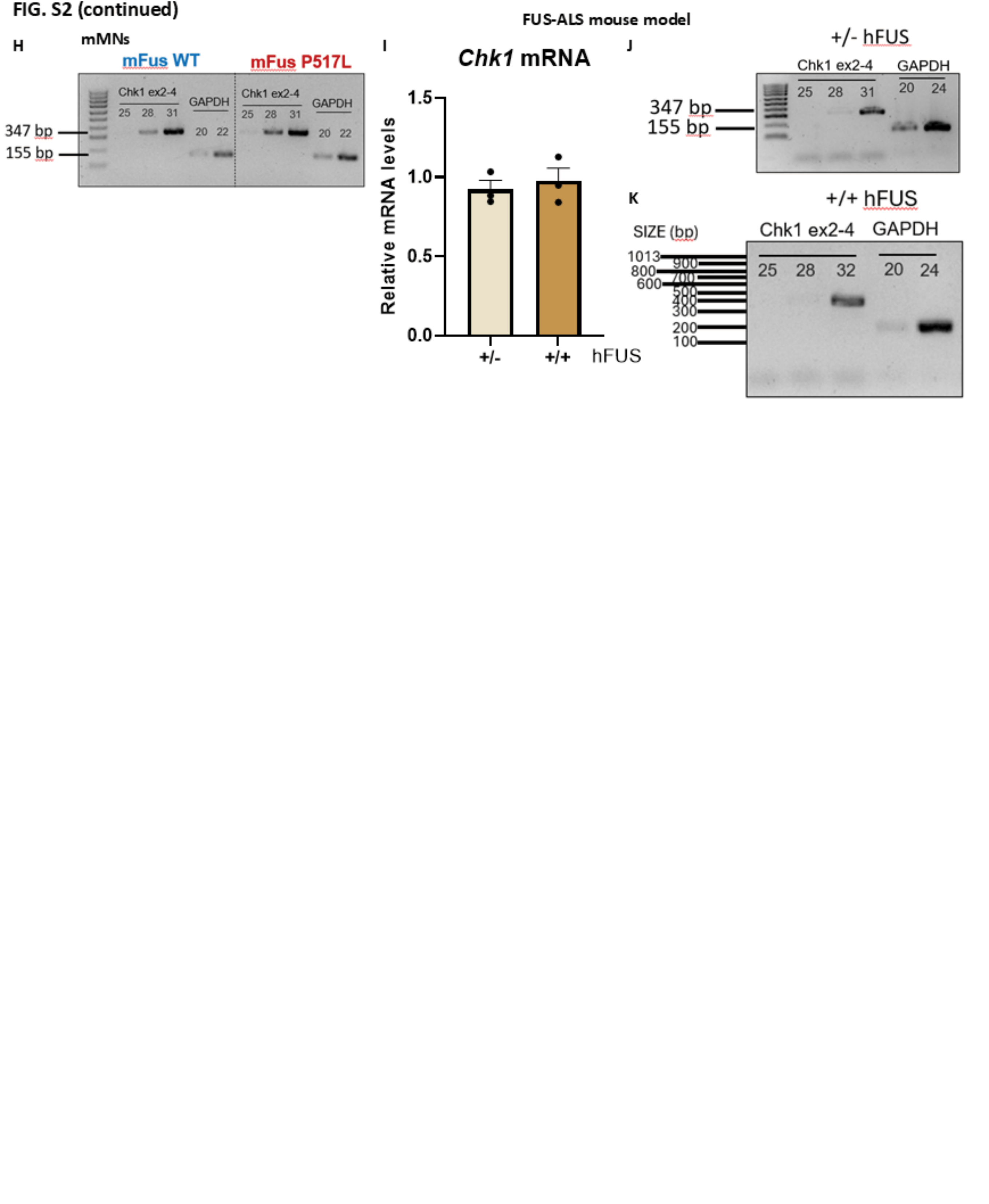

Supplement: Supplementary file 4 — Figure S2 (part 2) [file 41419_2026_8603_MOESM4_ESM.tif]

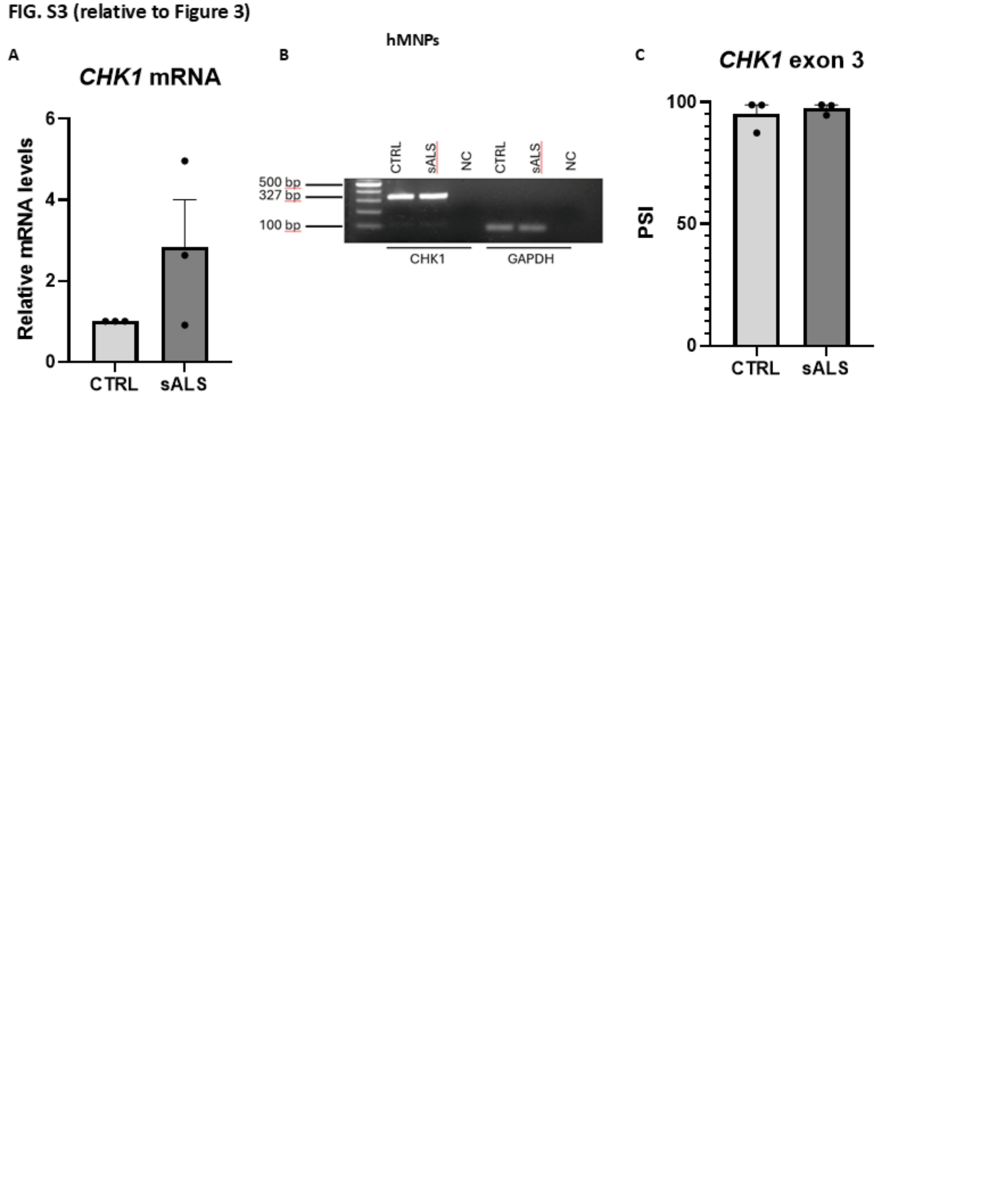

Supplement: Supplementary file 5 — Figure S3 [file 41419_2026_8603_MOESM5_ESM.tif]

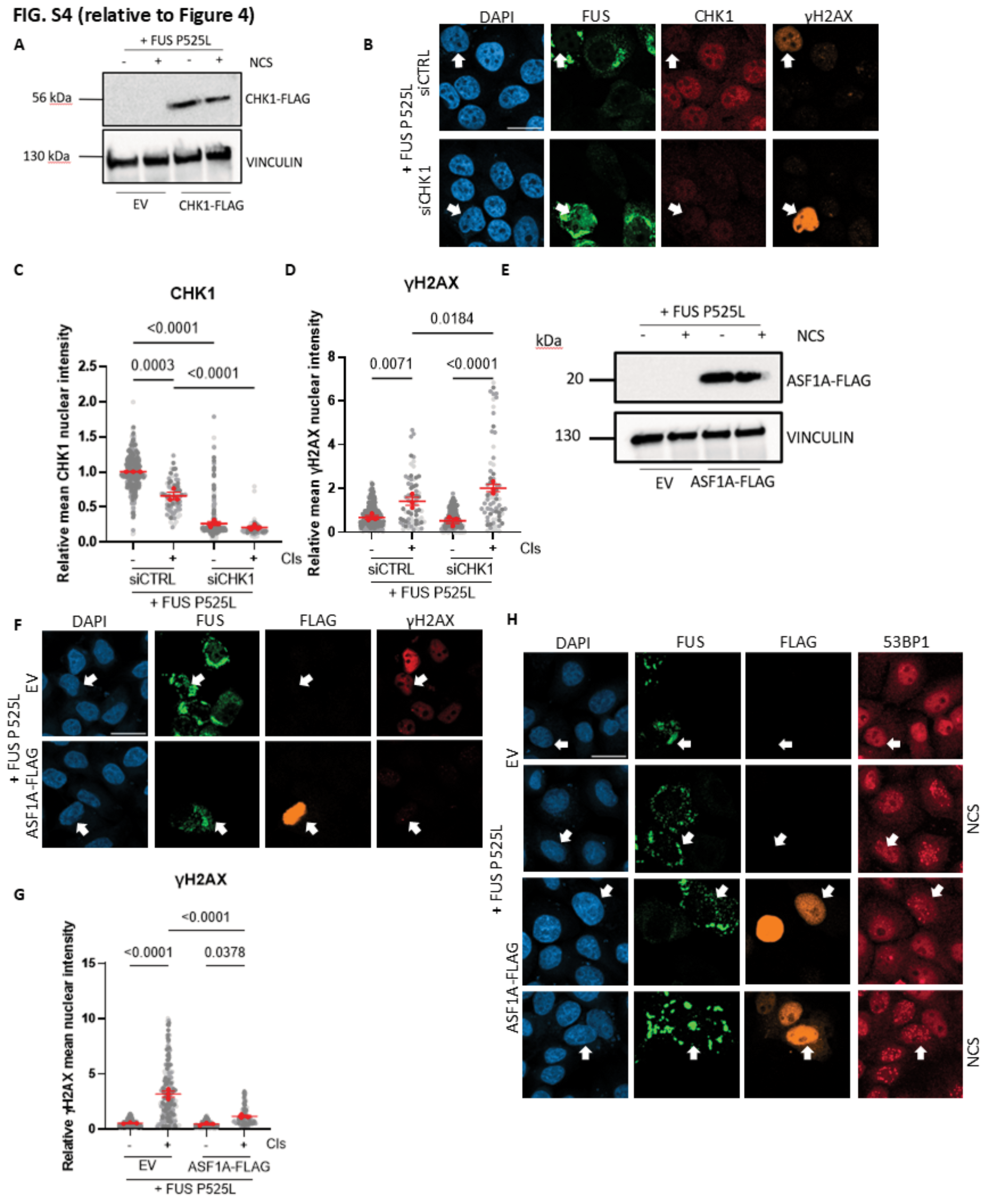

Supplement: Supplementary file 6 — Figure S4 (part 1) [file 41419_2026_8603_MOESM6_ESM.tif]

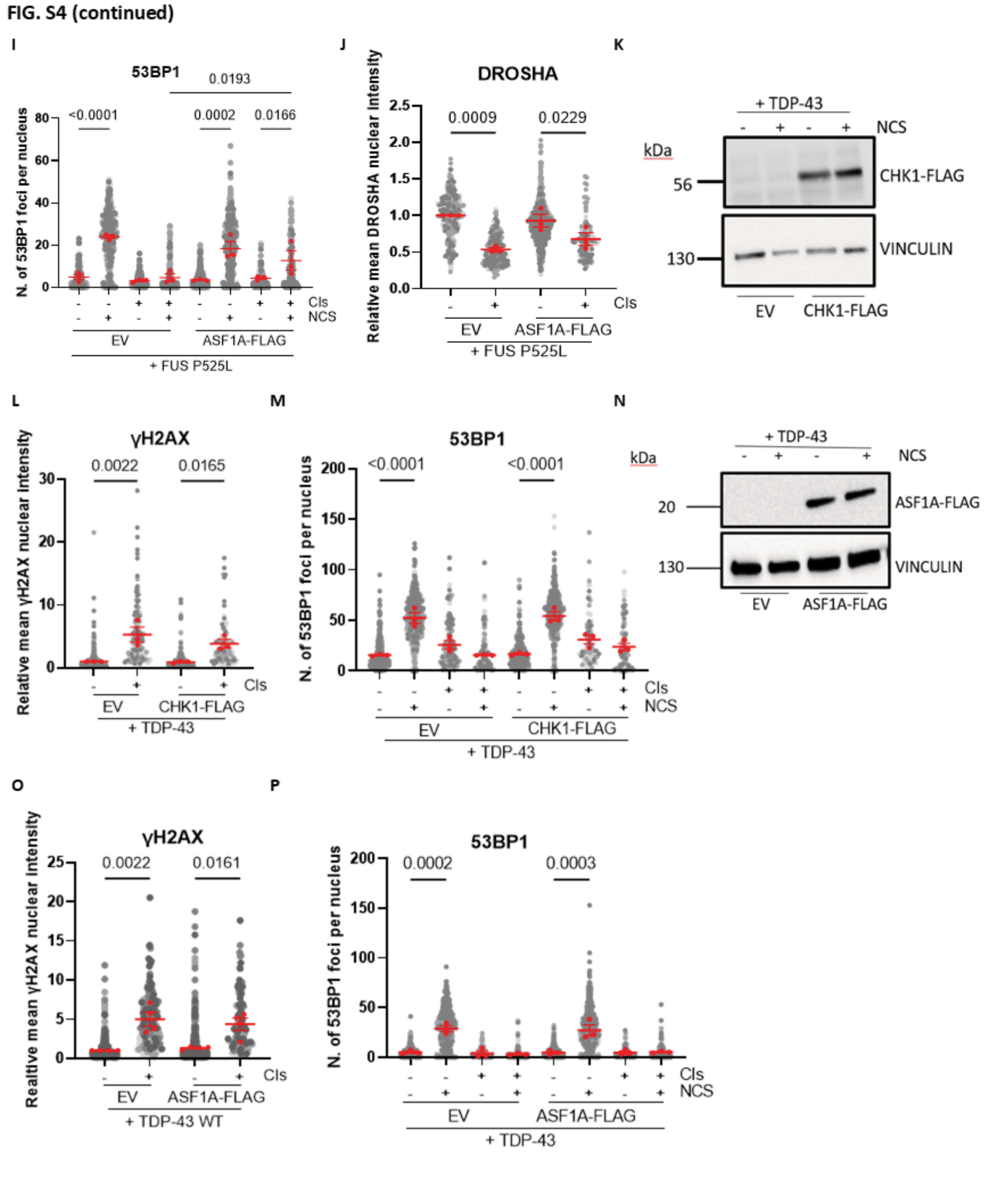

Supplement: Supplementary file 7 — Figure S4 (part 2) [file 41419_2026_8603_MOESM7_ESM.tif]

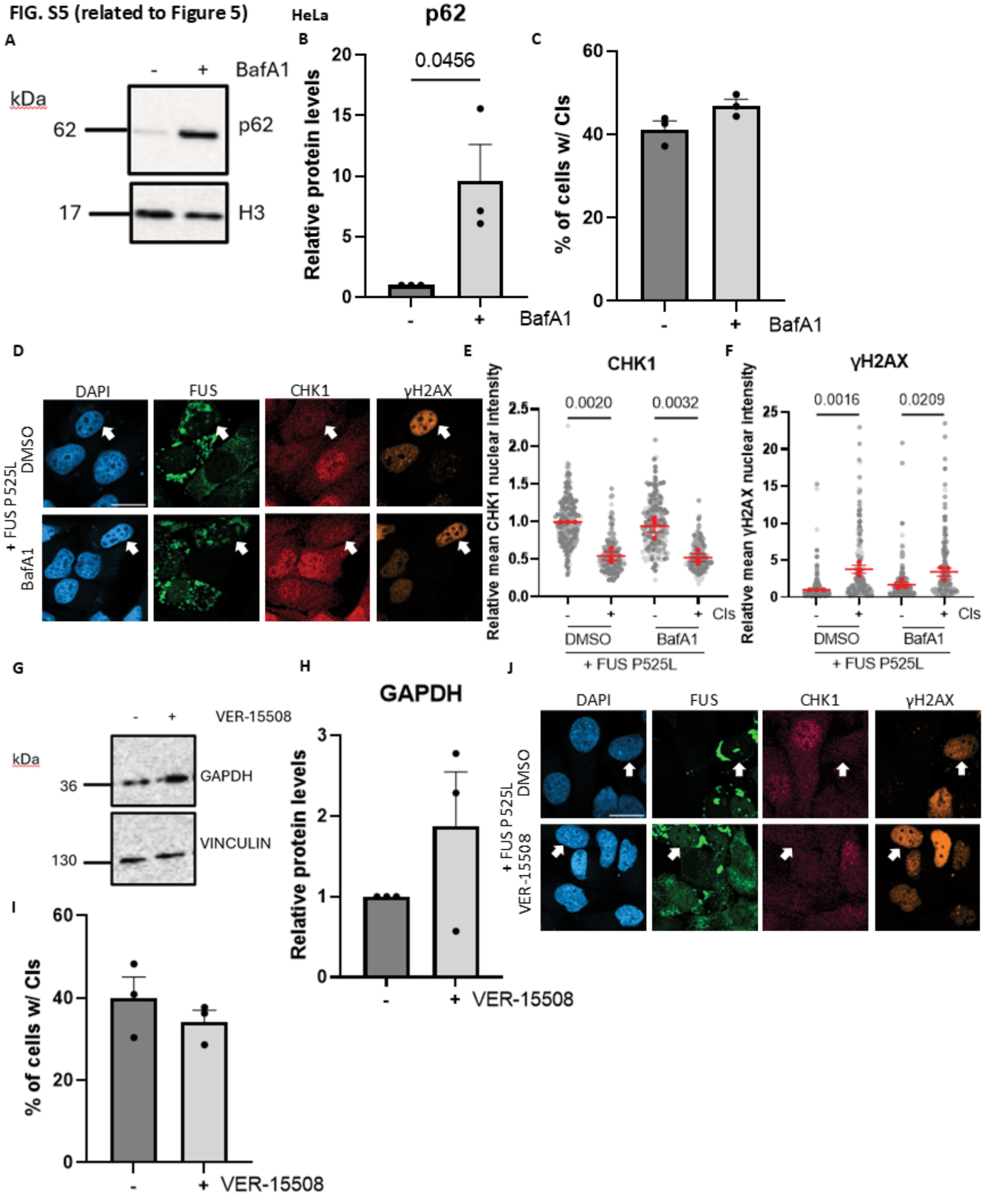

Supplement: Supplementary file 8 — Figure S5 (part 1) [file 41419_2026_8603_MOESM8_ESM.tif]

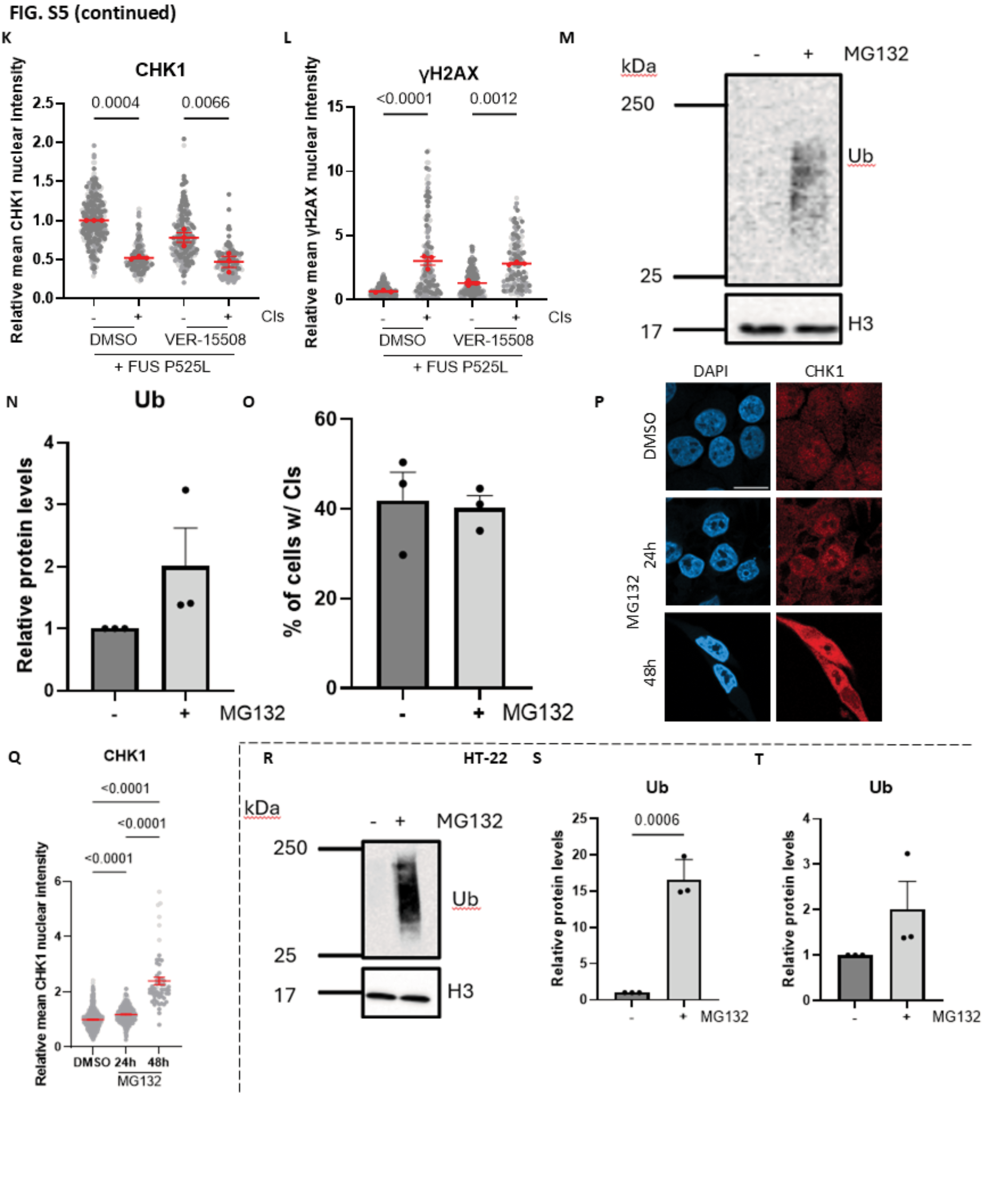

Supplement: Supplementary file 9 — Figure S5 (part 2) [file 41419_2026_8603_MOESM9_ESM.tif]
