# Supplementary material for: Proteasomal-dependent CHK1 degradation leads to DNA damage accumulation in ALS cellular model systems
Source: Cell Death Dis. 2026 May 6;17(1):599. doi: 10.1038/s41419-026-08603-6 (PMC13315745; doi:10.1038/s41419-026-08603-6)
Supplement: Supplementary file 10 — Original uncropped western blots and agarose gels [file 41419_2026_8603_MOESM10_ESM.pdf]

Dashed lines indicate where membranes were cropped

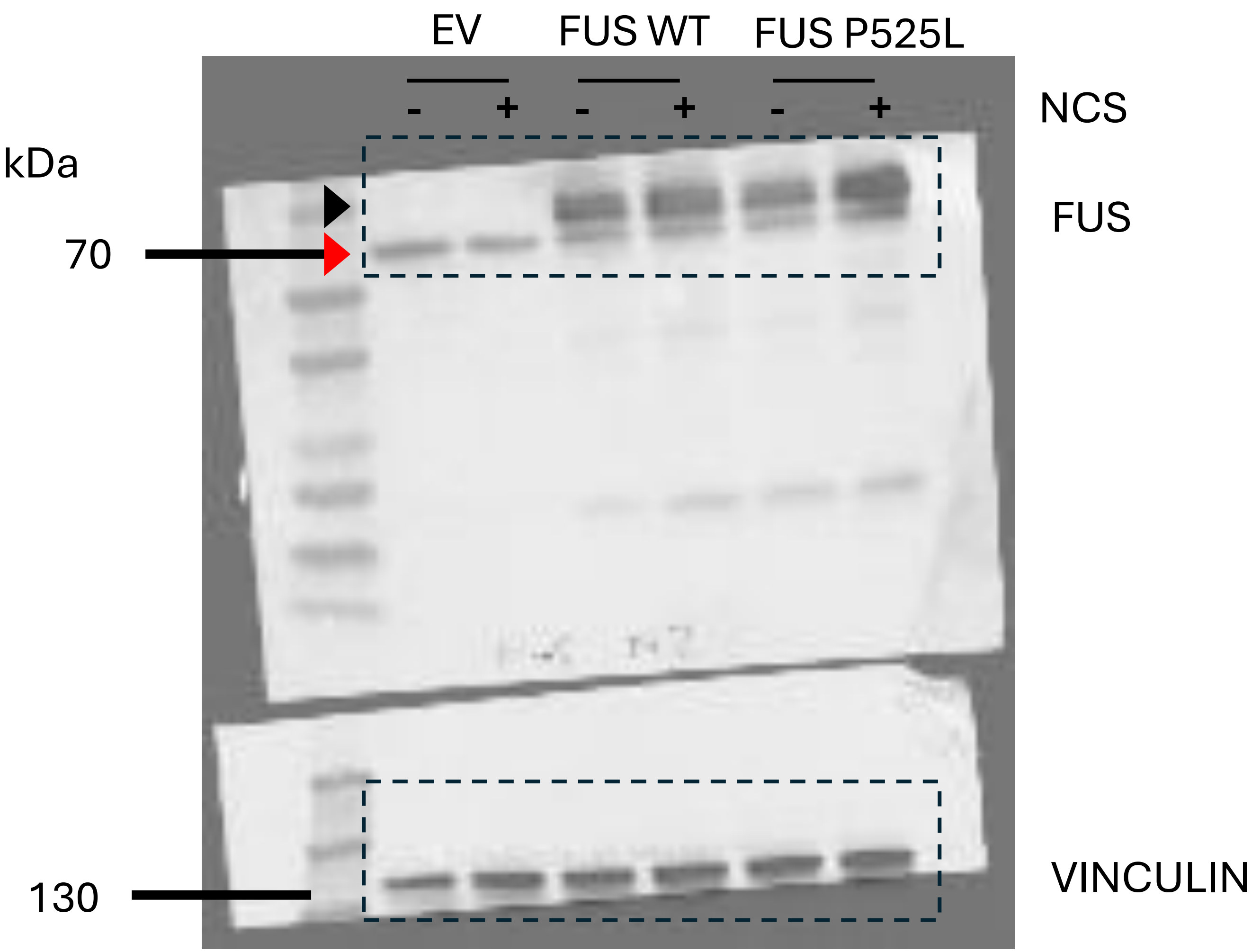

Relative to Fig. S1B: Representative western blot showing FUS overexpression levels in HeLa cells overexpressing WT FUS, mutant P525L FUS or transfected with an empty vector (EV) and probed for FUS treated or not with neocarzinostatin (NCS). The red arrowhead indicates the endogenous FUS protein, whereas the black arrowhead indicates the exogenous FUS protein. Vinculin was used as a loading control.

Dashed lines indicate where membranes were cropped

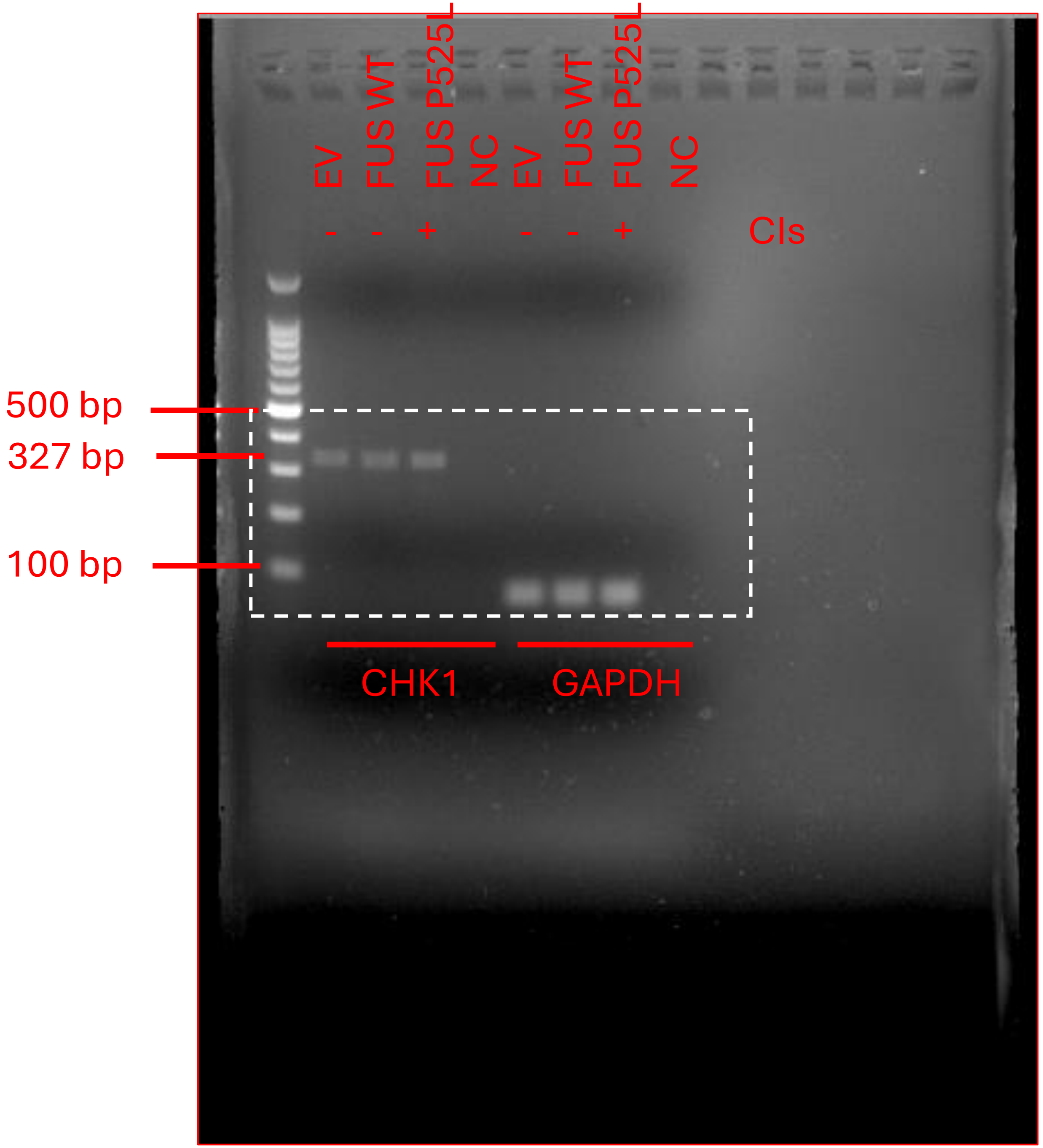

Relative to Fig. S1I: Representative agarose gel to investigate *CHK1* exon 3 splicing in HeLa cells overexpressing either WT or mutant P525L FUS or transfected with an EV. NC= negative control.

Dashed lines indicate where membranes were cropped

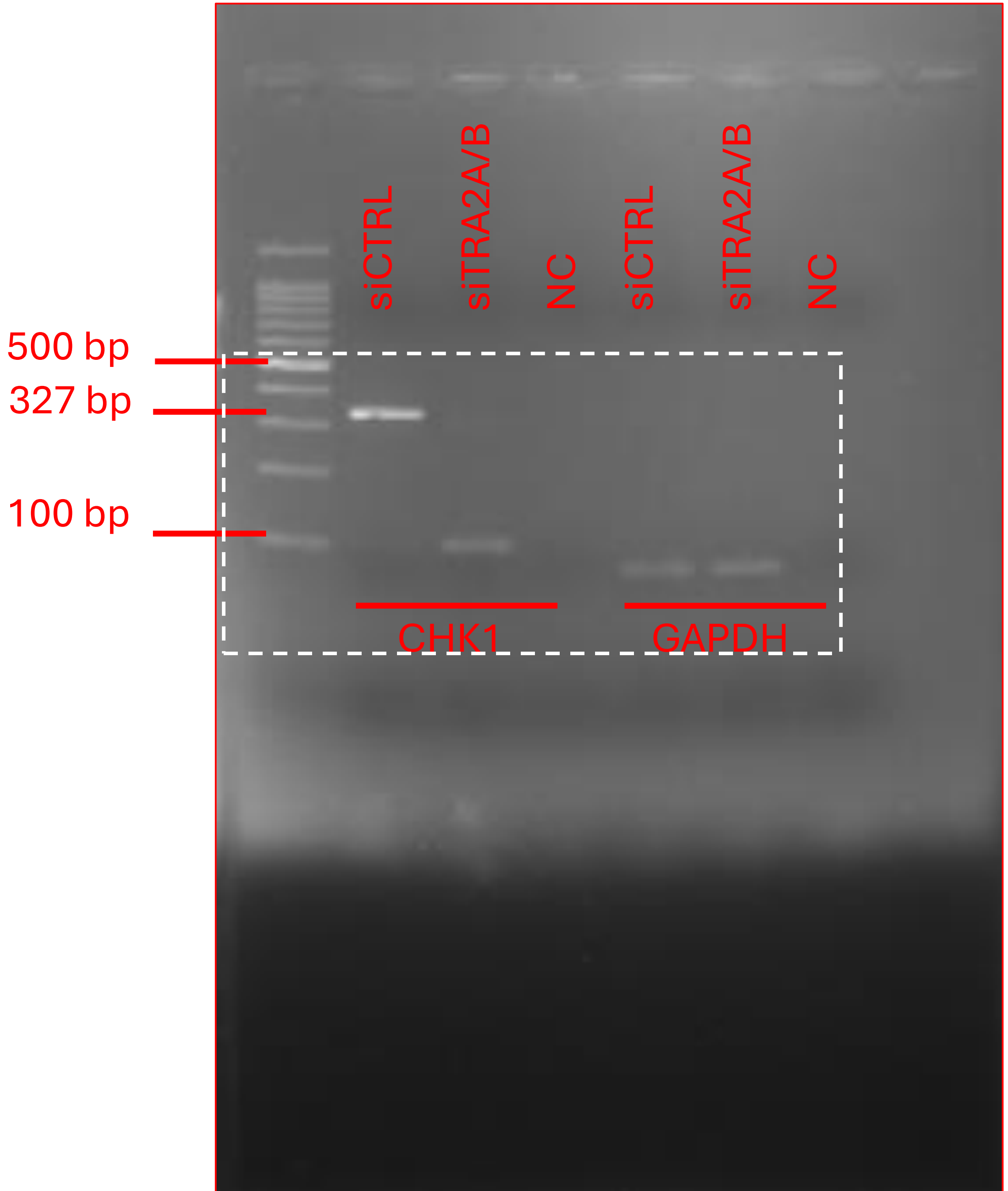

Relative to Fig. S1J: Representative agarose gel to investigate *CHK1* exon 3 splicing in HeLa cells transfected with a non-targeting control siRNA (siCTRL) or siRNAs targeting TRA2A/B (siTRA2A/B).

Dashed lines indicate where membranes were cropped

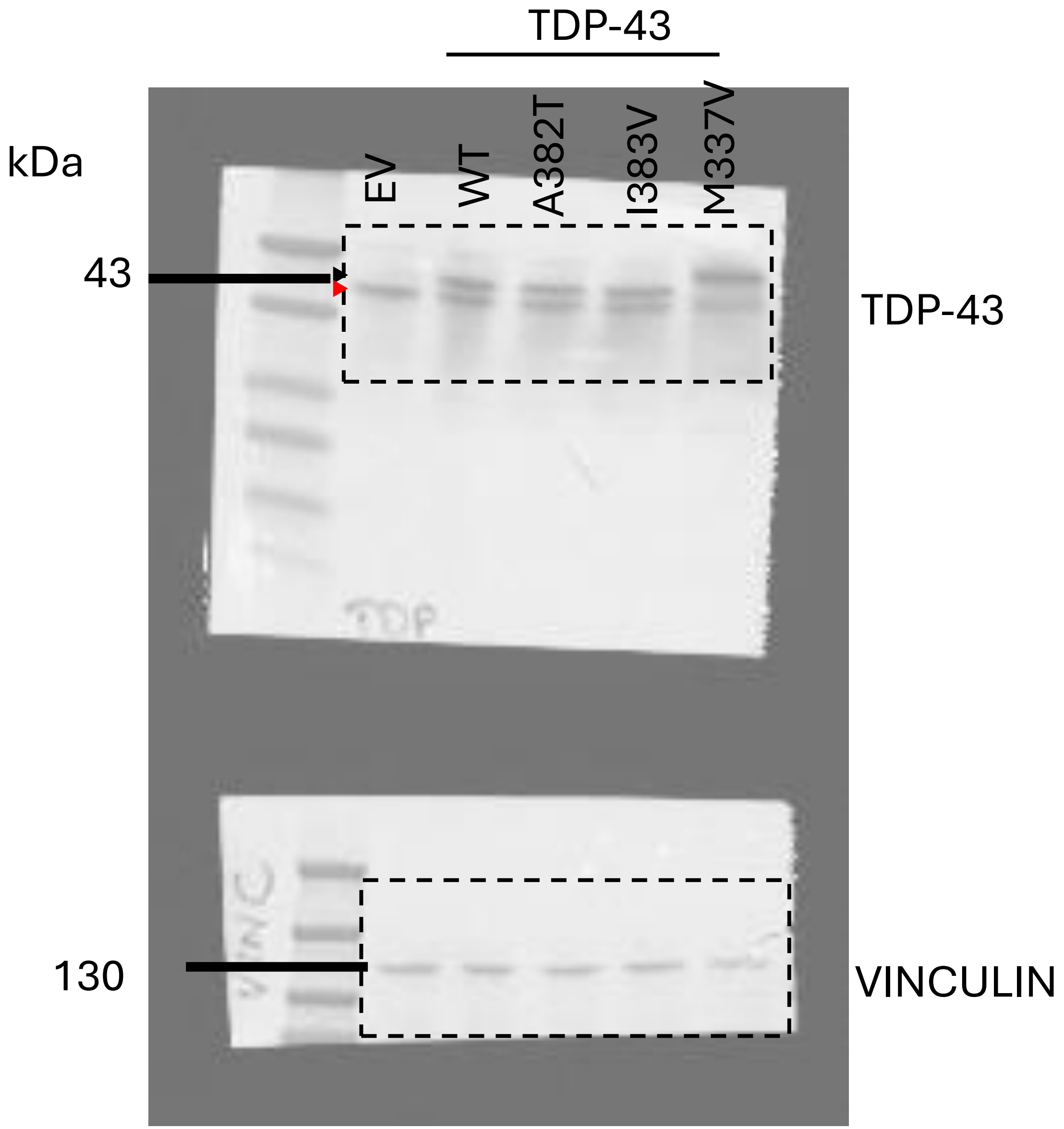

Relative to Fig. S1L: Representative western blot showing TDP-43 overexpression levels in HeLa cells overexpressing WT or three different ALS-associated TDP-43 mutants (A382T, I383V and M337V TDP-43) or transfected with an EV and probed for TDP-43. The red arrowhead indicates the endogenous TDP-43 protein, whereas the black arrowhead indicates the exogenous TDP-43 protein. Vinculin was used as a loading control.

Dashed lines indicate where membranes were cropped

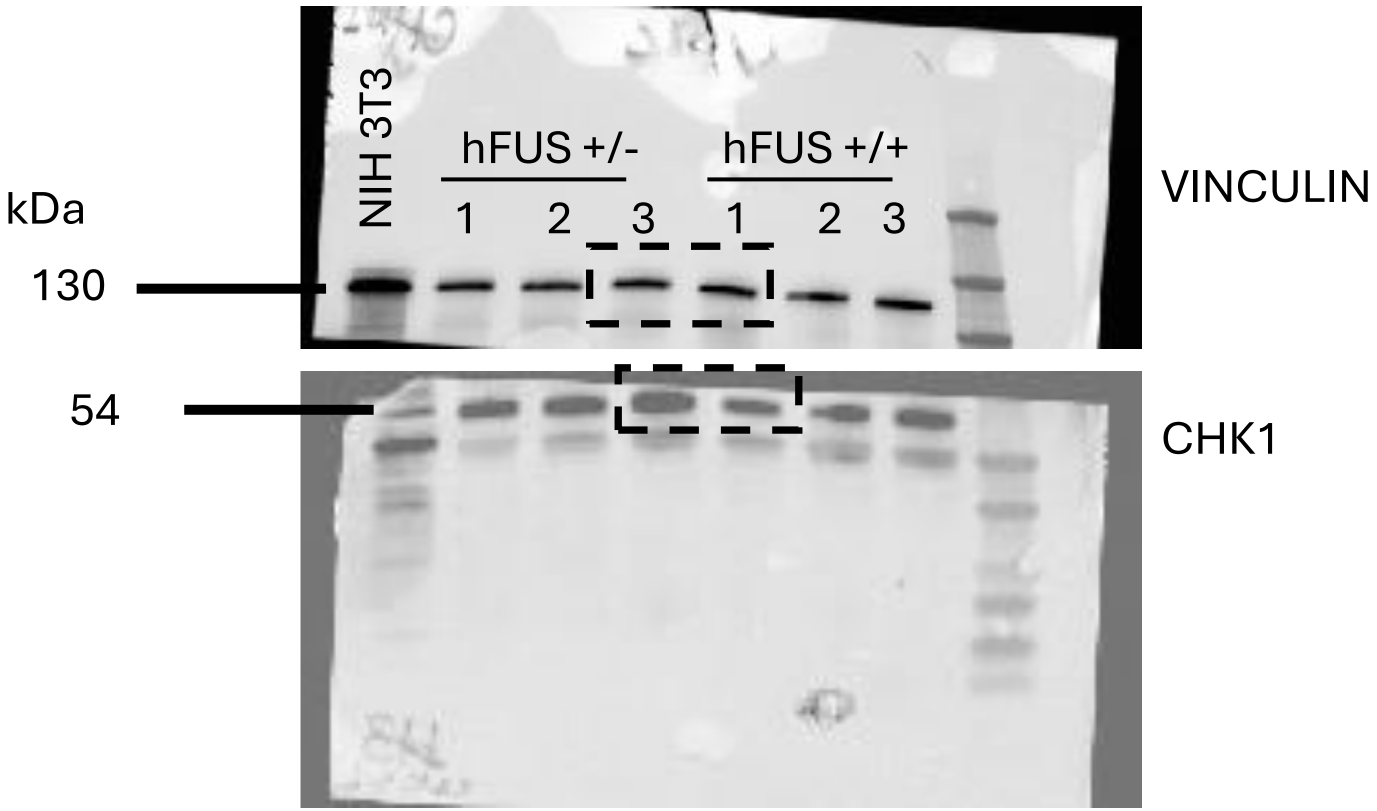

Relative to Fig. 2G: Representative western blot showing CHK1 protein levels in spinal cord samples derived from mice expressing human WT FUS (hFUS) in either heterozygosity (+/- hFUS) or homozygosity (+/+ hFUS). Vinculin was used as loading control.

Dashed lines indicate where membranes were cropped

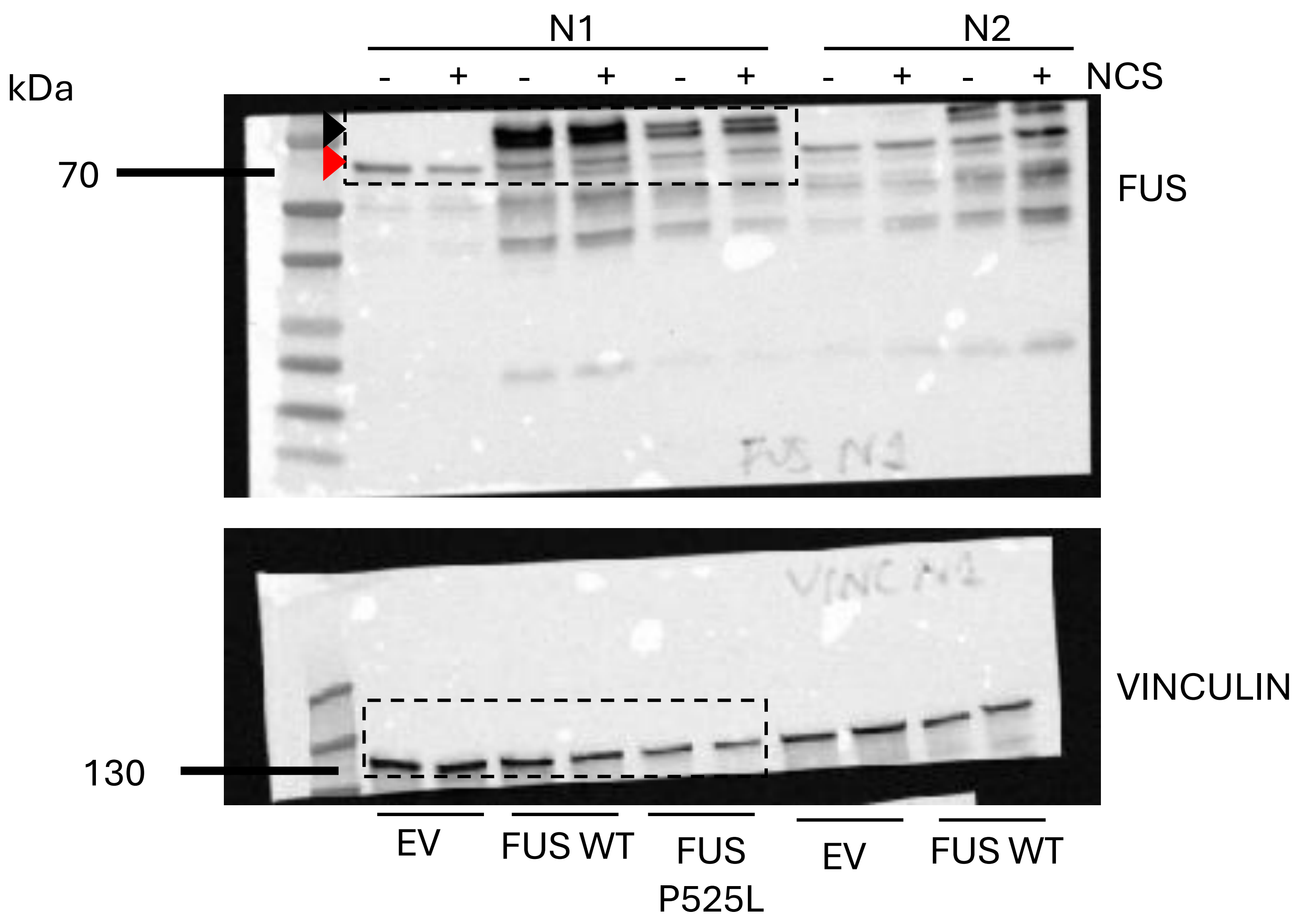

Relative to Fig. S2A: Representative western blot showing FUS overexpression levels in HT-22 cells overexpressing either WT or mutant P525L FUS or transfected with an EV and probed for FUS treated or not with NCS. Red and black arrowheads indicate the endogenous and exogenous FUS proteins, respectively. Vinculin was used as loading control.

Dashed lines indicate where membranes were cropped

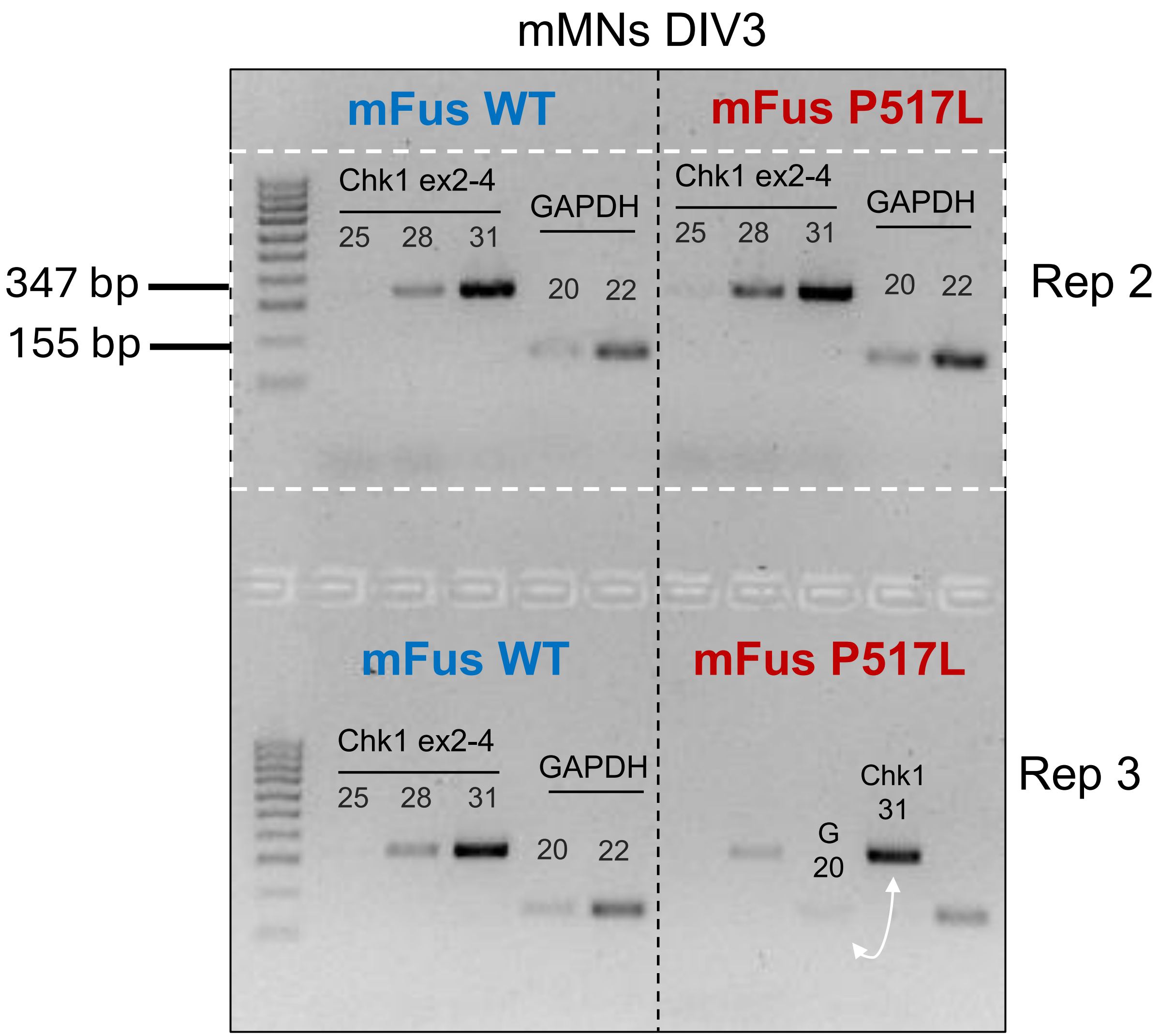

Relative to Fig. S2H: Representative agarose gel to investigate *Chk1* exon 3 splicing in murine motor neurons (mMNs) carrying either WT (mFus WT) or mutant (mFus P517L) murine *Fus* gene. The numbers on the gel indicate the PCR cycles after which samples were collected.

Dashed lines indicate where membranes were cropped

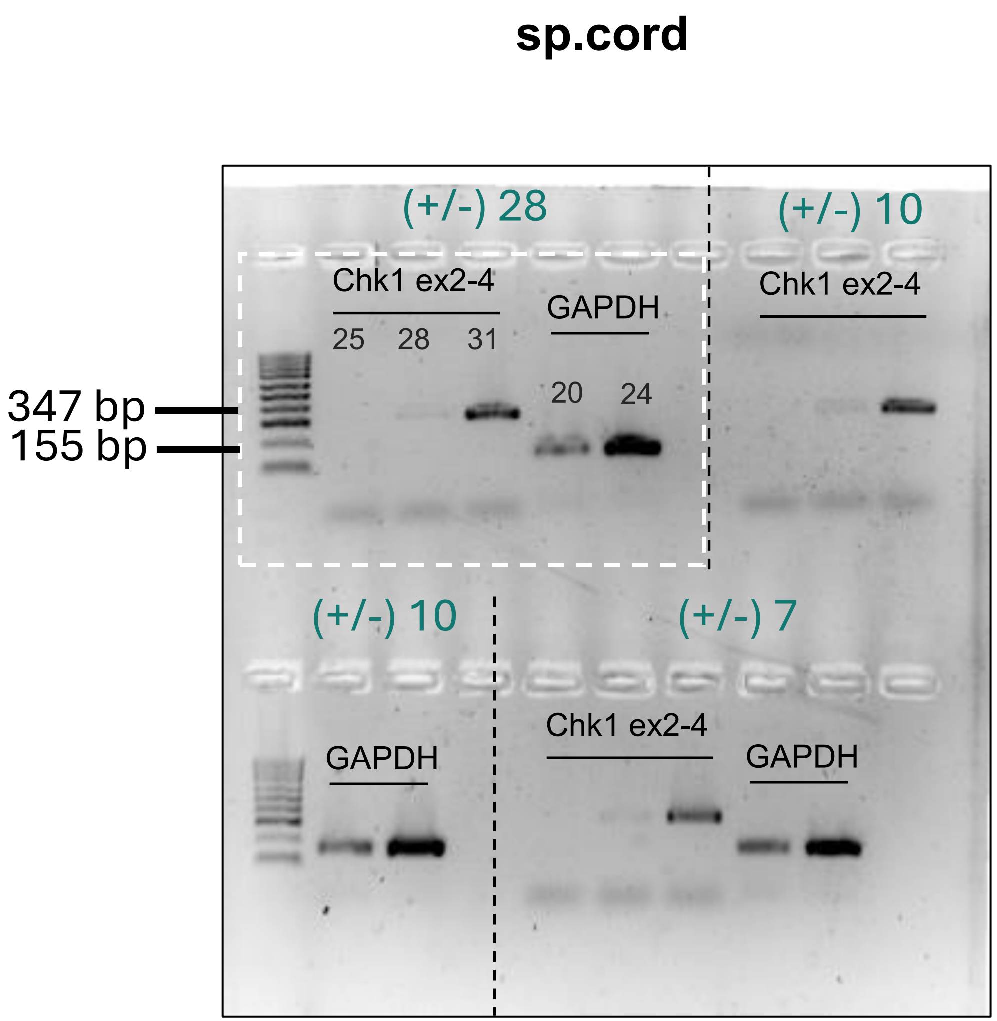

Relative to Fig. S2J: Representative agarose gel to investigate *Chk1* exon 3 splicing in spinal cord samples derived from mice expressing human WT FUS (hFUS) in heterozygosity (+/- hFUS). The numbers on the gel indicate the PCR cycles after which samples were collected.

Dashed lines indicate where membranes were cropped

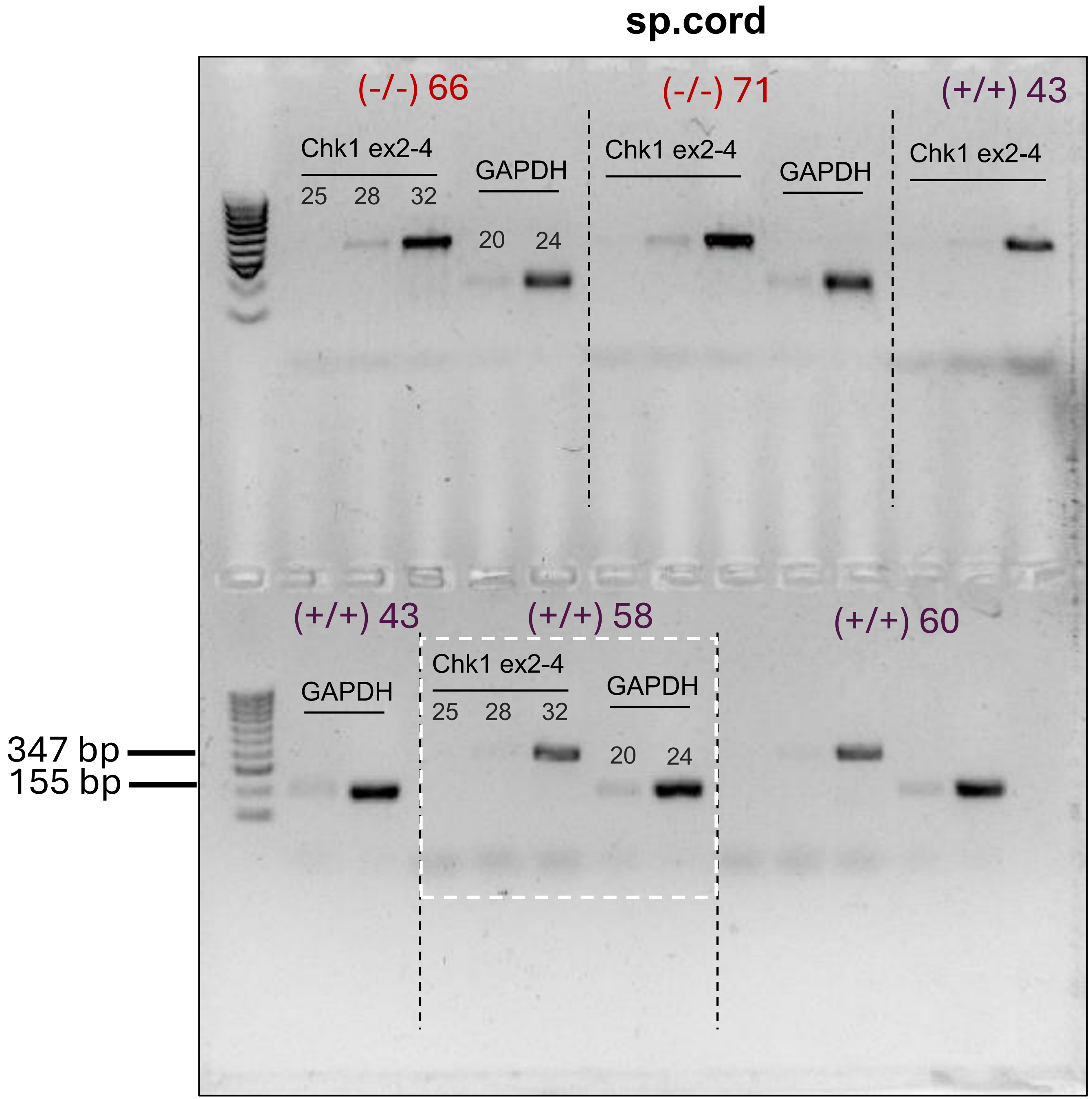

Relative to Fig. S2K: Representative agarose gel to investigate *Chk1* exon 3 splicing in spinal cord samples derived from mice expressing human WT FUS (hFUS) in homozygosity (+/+ hFUS). The numbers on the gel indicate the PCR cycles after which samples were collected.

Dashed lines indicate where membranes were cropped

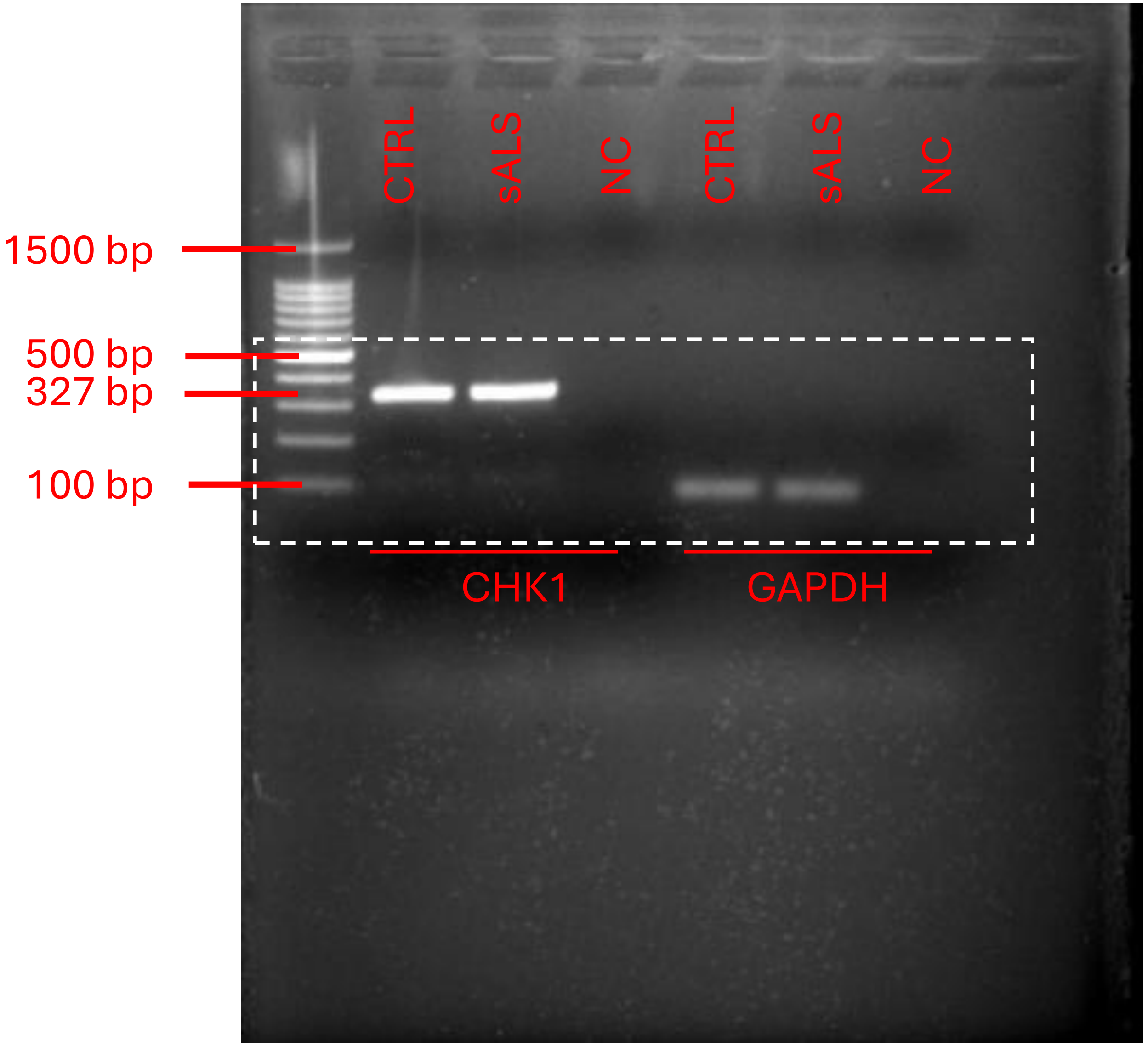

Relative to Fig. S3B: Representative agarose gel to investigate *CHK1* exon 3 splicing in human motor neuron progenitors (hMNPs) derived from a healthy control (CTRL) or from a sporadic ALS patient (sALS).

<u>Dashed lines indicate where membranes were cropped</u>

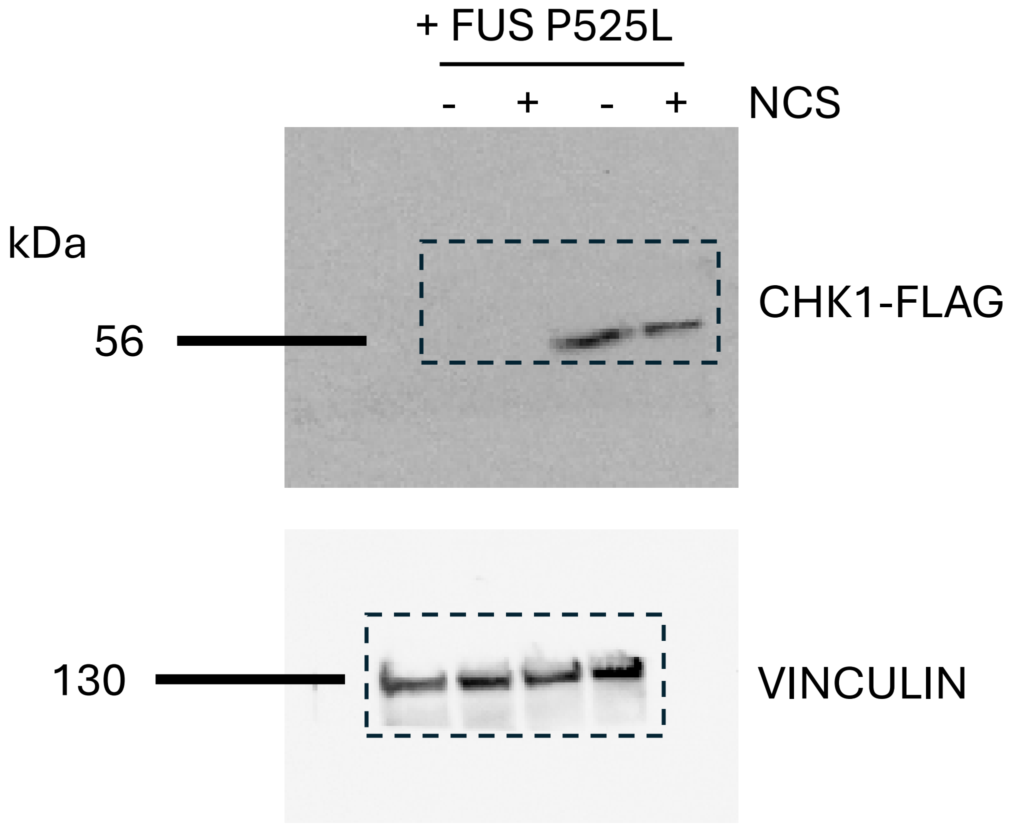

Relative to Fig.S4A: Representative western blot showing CHK1 overexpression levels in HeLa cells overexpressing mutant P525L FUS together with a plasmid encoding for FLAG-tagged CHK1 (CHK1-FLAG) or an EV, treated or not with NCS and probed for FLAG. Vinculin was used as loading control.

Dashed lines indicate where membranes were cropped

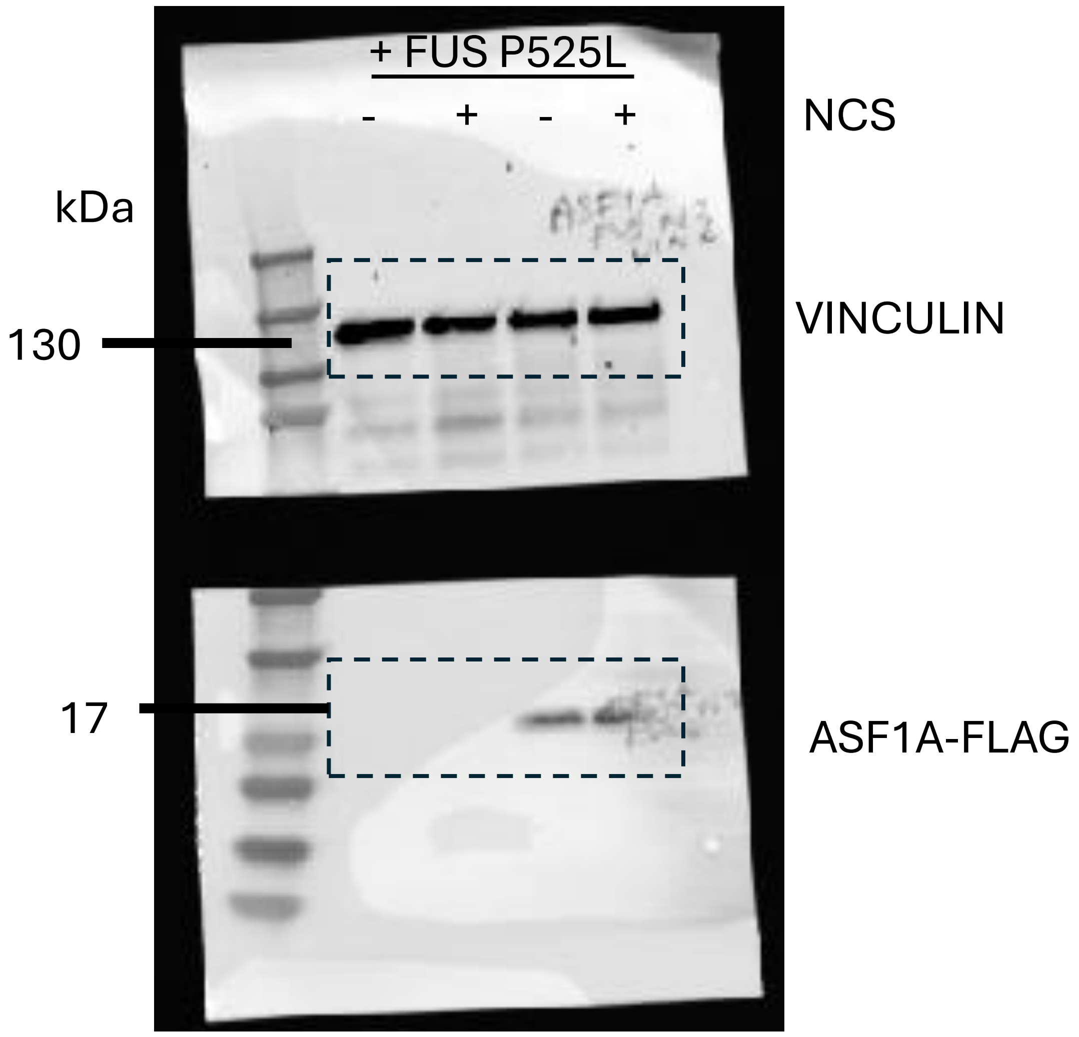

Relative to Fig. S4E: Representative western blot showing ASF1A overexpression levels in HeLa cells overexpressing mutant P525L FUS together with a plasmid encoding for FLAG-tagged ASF1A (ASF1A-FLAG) or an EV, treated or not with NCS and probed for FLAG. Vinculin was used as loading control.

Dashed lines indicate where membranes were cropped

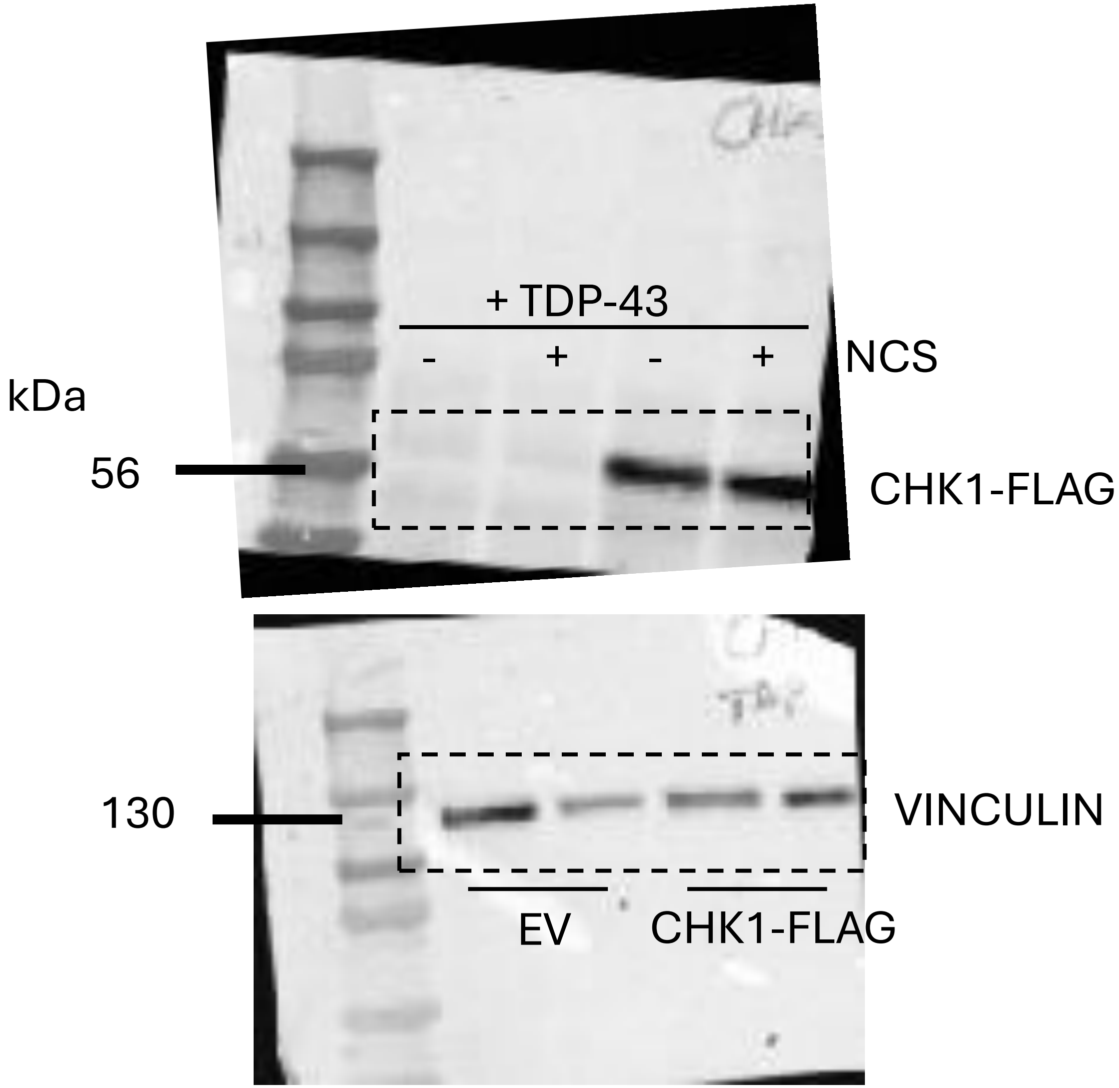

Relative to Fig. S4K: Representative western blot showing CHK1 overexpression levels in HeLa cells overexpressing TDP-43 together with CHK1-FLAG or an EV, treated or not with NCS and probed for FLAG. Vinculin was used as loading control.

Dashed lines indicate where membranes were cropped

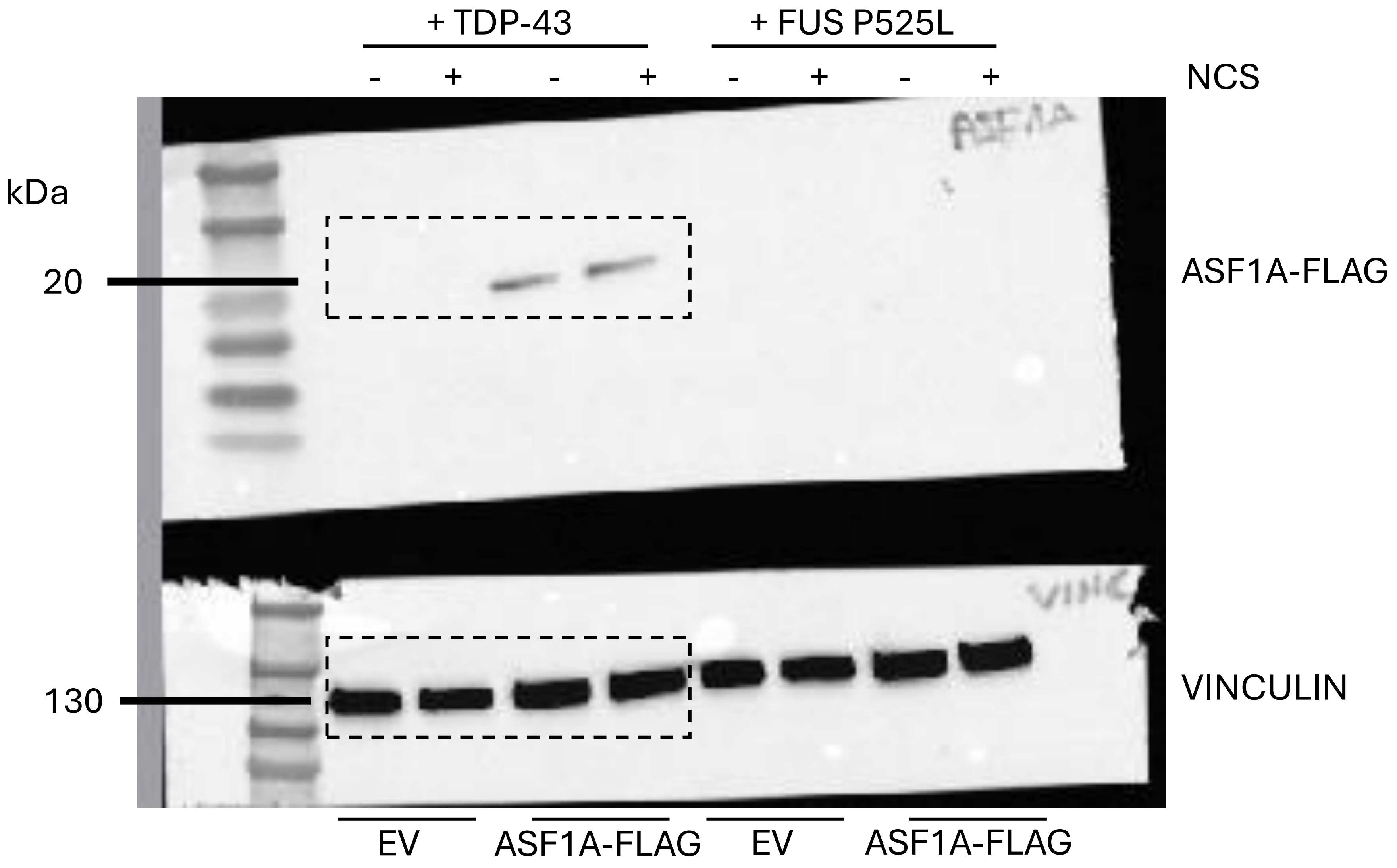

Relative to Fig. S4N: Representative western blot showing ASF1A overexpression levels in HeLa cells overexpressing TDP-43 together with ASF1A-FLAG or an EV, treated or not with NCS and probed for FLAG. Vinculin was used as loading control.

Dashed lines indicate where membranes were cropped

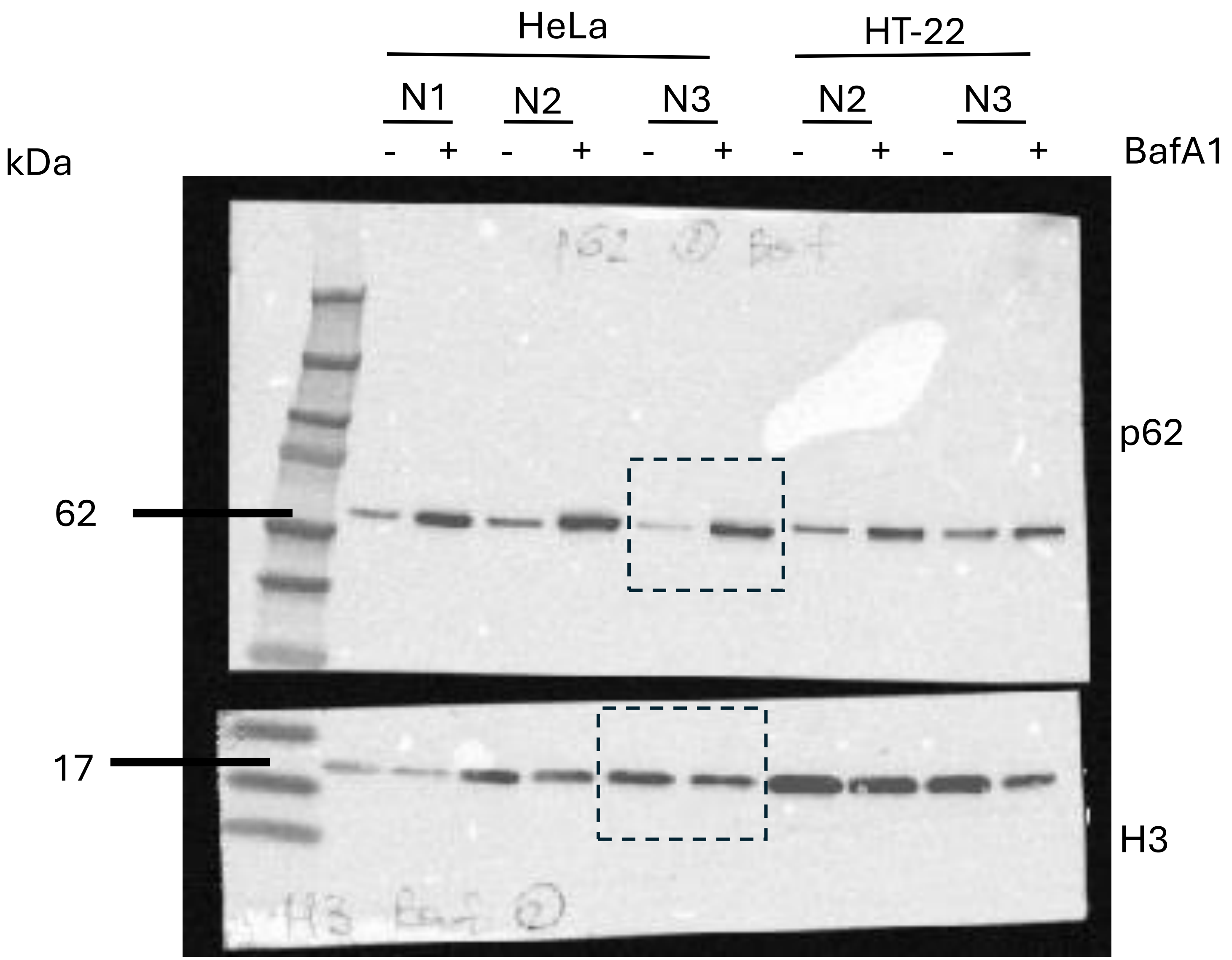

Relative to Fig. S5A: Representative western blot showing p62 protein levels in HeLa cells overexpressing mutant P525L FUS and treated or not with the autophagy inhibitor bafilomycin A1 (BafA1). H3 was used as loading control.

Dashed lines indicate where membranes were cropped

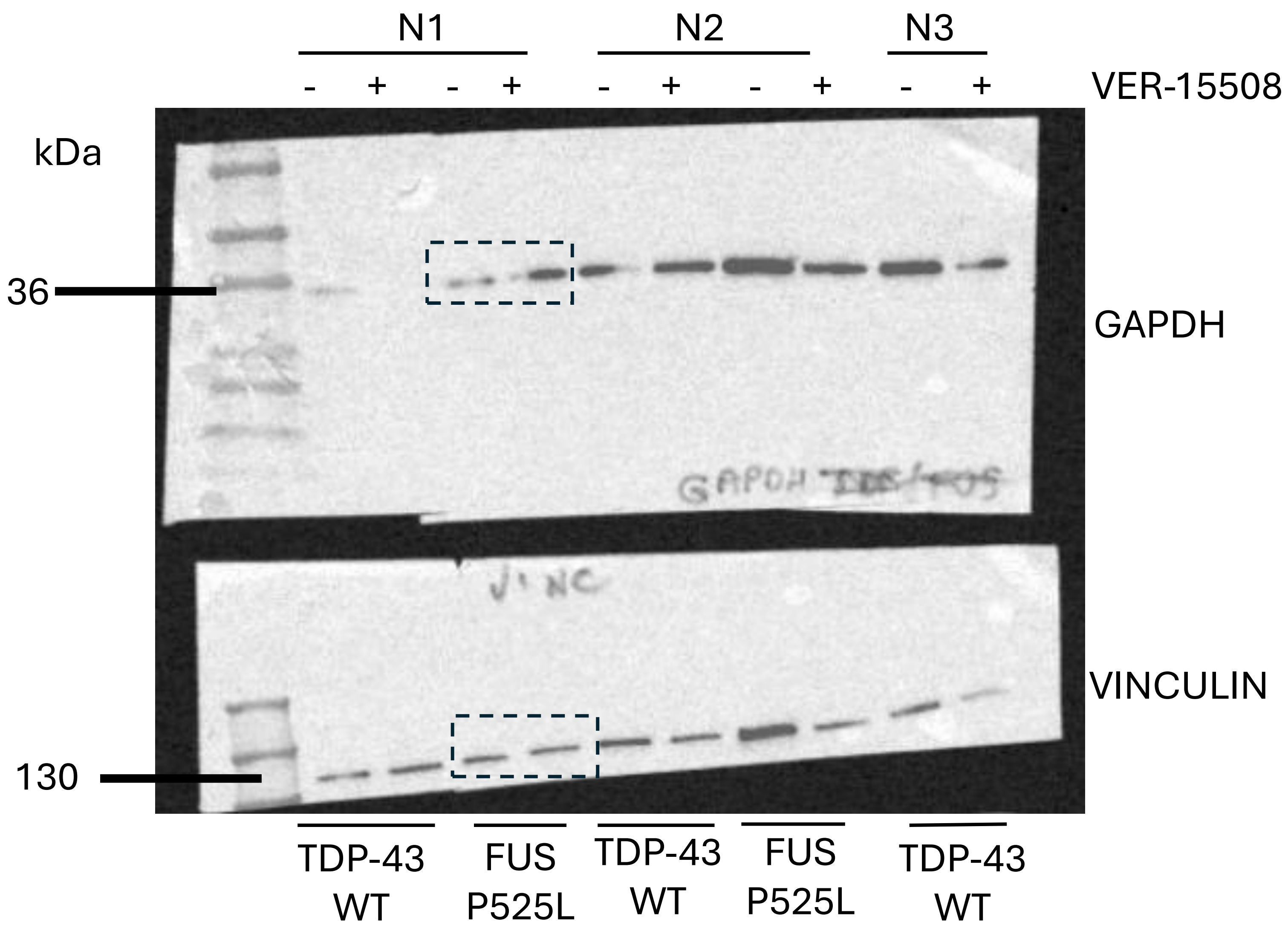

Relative to Fig. S5G: Representative western blot showing GAPDH protein levels in HeLa cells overexpressing mutant P525L FUS and treated or not with the HSC70 inhibitor VER-15508. Vinculin was used as loading control.

Dashed lines indicate where membranes were cropped

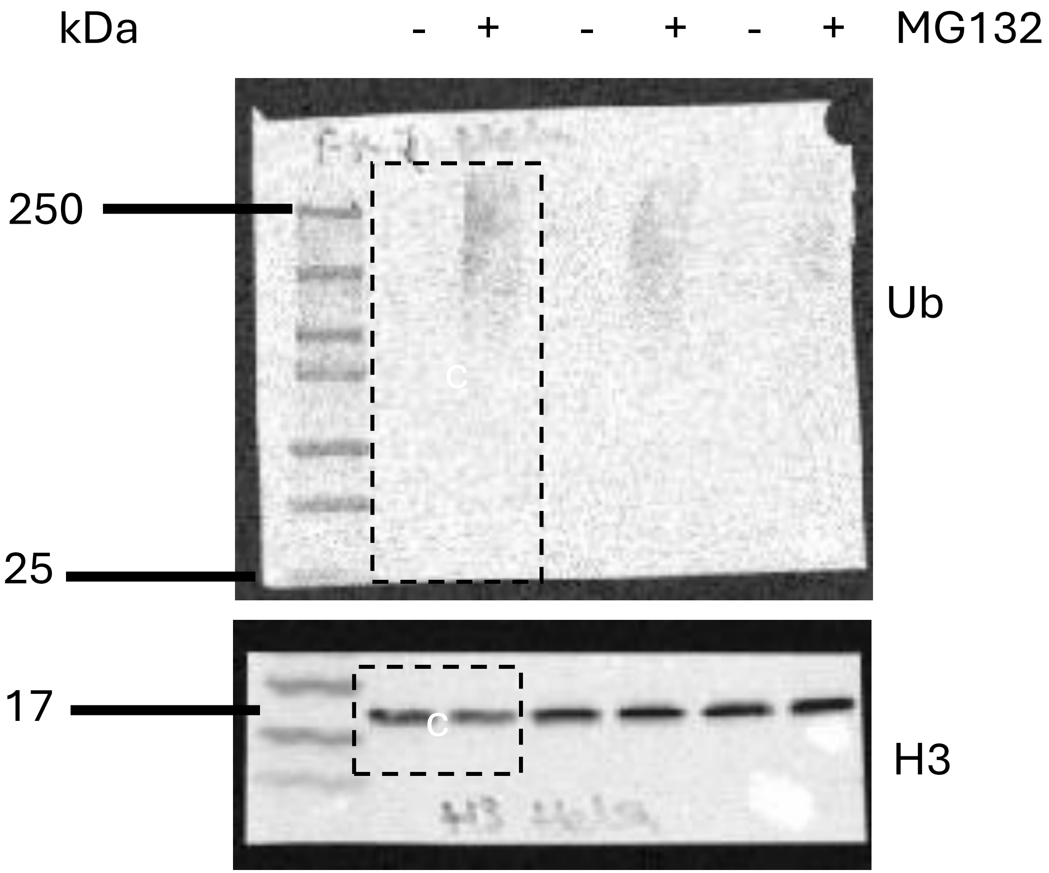

Relative to Fig. S5M: Representative western blot showing the accumulation of ubiquitylated proteins in HeLa cells overexpressing mutant P525L FUS and treated or not with the proteasome inhibitor MG132. H3 was used as loading control.

Dashed lines indicate where membranes were cropped

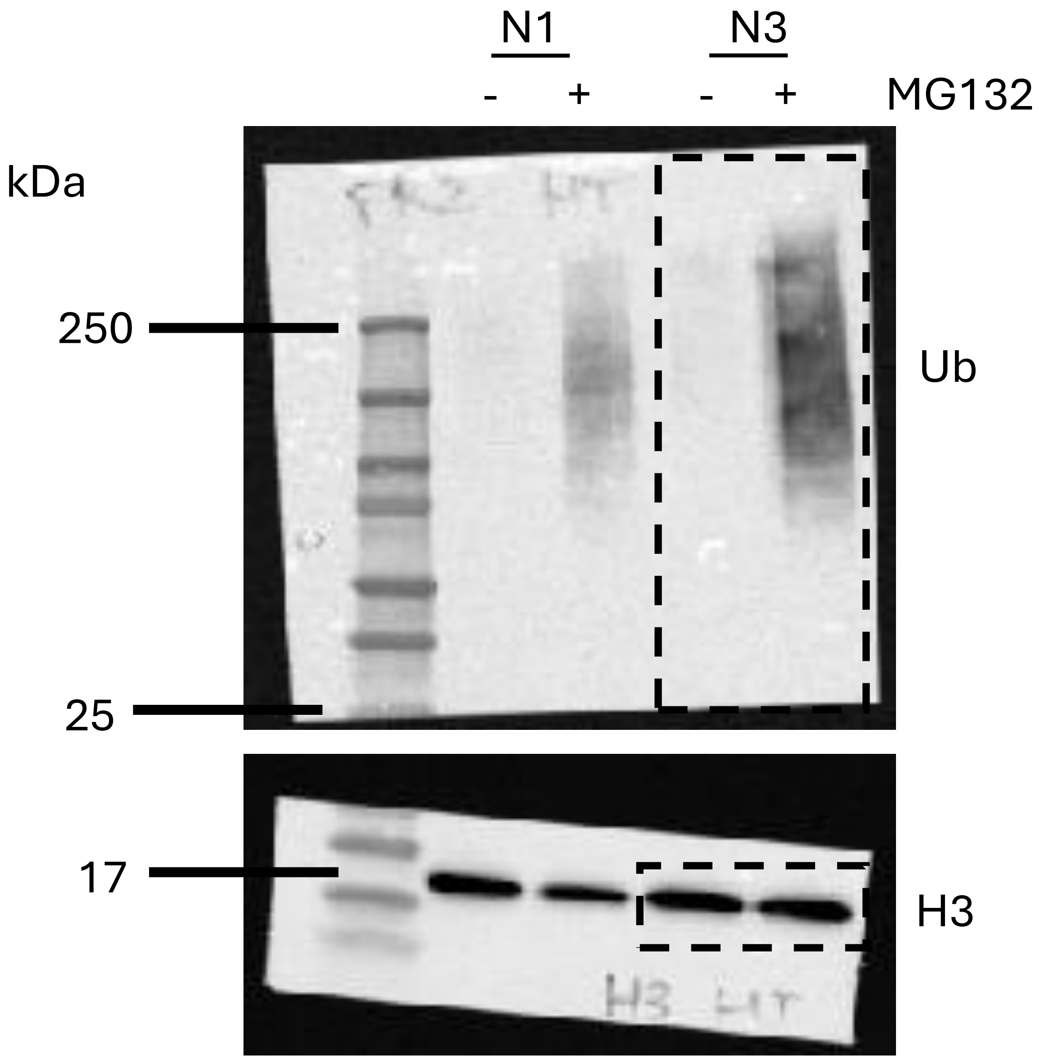

Relative to Fig. S5R: Representative western blot showing the accumulation of ubiquitylated proteins in HT-22 cells overexpressing mutant P525L FUS and treated or not with the proteasome inhibitor MG132. H3 was used as loading control.
