# Supplementary material for: Proteasomal-dependent CHK1 degradation leads to DNA damage accumulation in ALS cellular model systems
Source: Cell Death Dis. 2026 May 6;17(1):599. doi: 10.1038/s41419-026-08603-6 (PMC13315745; doi:10.1038/s41419-026-08603-6)
Supplement: Supplementary file 11 — Supplementary figures legends [file 41419_2026_8603_MOESM11_ESM.docx]

**Figure S1 (relative to Figure 1):** **A:** Schematic representation of the constructs used to overexpress either WT or mutant P525L mCerulean (mCer)-tagged or mVenus (mVen)-tagged FUS. The numbers indicate amino acid residues whereas the black stars indicate the position of the P525L FUS mutation. N-term= N-terminus, C-term= C-terminus. For further information regarding the plasmids used in this study see the “Material and methods” section. **B:** Representative western blot showing FUS overexpression levels in HeLa cells overexpressing WT FUS, mutant P525L FUS or transfected with an EV and probed for FUS treated or not with neocarzinostatin (NCS). The red arrowhead indicates the endogenous FUS protein, whereas the black arrowhead indicates the exogenous FUS protein. Vinculin was used as a loading control. **C:** Quantification of FUS overexpression levels in cells treated as in B. Error bars indicate mean ± SEM. N= three independent experiments. **D:** Quantification of the percentage of cells bearing CIs in cells treated as in B. Error bars indicate mean ± SEM. N= three independent experiments. **E:** Quantification of γH2AX nuclear intensity in HeLa cells overexpressing either WT or mutant P525L FUS or transfected with an EV. Red dot and error bars are mean ± SEM. N= three independent experiments. At least 50 cells per condition were analysed. **F:** Quantification of the number of 53BP1 foci per nucleus in HeLa cells treated as in B. Red dot and error bars are mean ± SEM. N= three independent experiments. At least 50 cells per condition were analysed. **G:** Quantification of *CHK1* mRNA levels in HeLa cells treated as in E. Error bars are mean ± SEM. N= seven independent experiments. **H:** Quantification of *ASF1A* mRNA levels in cells treated as in E. Error bars are mean ± SEM. N= seven independent experiments. **I:** Representative agarose gel to investigate CHK1 exon 3 splicing in HeLa cells treated as in E. NC= negative control. **J:** Representative agarose gel to investigate CHK1 exon 3 splicing in HeLa cells transfected with a non-targeting control siRNA (siCTRL) or siRNAs targeting TRA2A/B (siTRA2A/B). **K:** Schematic representation of the constructs used to overexpress either WT or mutant A382T and I383V Myc-tagged TDP-43 or the FLAG-tagged mutant M337V TDP-43. The numbers indicate amino acid residues whereas the black, white and light blue stars indicate the position of the A382T, I383V and M337V TDP-43 mutations, respectively. N-term= N-terminus, C-term= C-terminus. For further information regarding the plasmids used in this study see the “Material and methods” section.  **L:** Representative western blot showing TDP-43 overexpression levels in HeLa cells overexpressing WT TDP-43, three different ALS-associated TDP-43 mutants (A382T, I383V and M337V TDP-43) or transfected with an EV and probed for TDP-43. The red arrowhead indicates the endogenous TDP-43 protein, whereas the black arrowhead indicates the exogenous TDP-43 protein. Vinculin was used as a loading control. **M:** Quantification of TDP-43 overexpression levels in cells treated as in L. Error bars indicate mean ± SEM. N= three independent experiments. **N:** Representative images of HeLa cells treated as in L and probed for TDP-43, CHK1 and γH2AX. DNA was counterstained with DAPI. White arrows indicate cells overexpressing either WT or mutant TDP-43 bearing CIs whereas yellow arrows indicate cells overexpressing either WT or mutant TDP-43 lacking CIs. Scale bar is 20 µm. **O:** Quantification of CHK1 protein nuclear intensity in cells treated as in L. Red dots and error bars are mean ± SEM. N= three independent experiments. At least 40 cells per condition were analysed. **P:** Quantification of γH2AX nuclear intensity in cells treated as in L. Red dots and error bars are mean ± SEM. N= three independent experiments. At least 40 cells per condition were analysed.

**Figure S2 (relative to Figure 2): A:** Representative western blot showing FUS overexpression levels in HT-22 cells overexpressing either WT or mutant P525L FUS or transfected with an EV and probed for FUS treated or not with NCS. Red and black arrowheads indicate the endogenous and exogenous FUS proteins, respectively. Vinculin was used as loading control. **B:** Quantification of FUS overexpression levels in cells treated as in A. Error bars are mean ± SEM. N= three independent experiments. **C:** Quantification of the percentage of cells bearing CIs in HT-22 cells treated as in A. Error bars are mean ± SEM. N= three independent experiments. **D:** Quantification of γH2AX nuclear intensity in HT-22 overexpressing either WT or mutant P525L FUS or transfected with an EV. Red dots and error bars are mean ± SEM. N= three independent experiments. At least 50 cells per condition were analysed. **E:** Representative images of HT-22 treated as in A and probed for FUS and 53BP1. DNA was counterstained with DAPI. White arrows indicate representative cells showing the described phenotype. Scale bar is 20 µm. **F:** Quantification of the number of 53BP1 foci per nucleus in cells treated as in A. Red dots and error bars are mean ± SEM. N= three independent experiments. At least 50 cells per condition were analysed. **G:** Quantification of *Chk1* mRNA levels in mMNs carrying either WT (mFus WT) or mutant (mFus P517L) murine *Fus* gene. Error bars are mean ± SEM. N= three independent experiments. **H:** Representative agarose gel to investigate CHK1 exon 3 splicing in mMNs carrying either WT (mFus WT) or mutant (mFus P517L) murine *Fus* gene. The numbers on the gel indicate the PCR cycles after which samples were collected. **I:** Quantification of *Chk1* mRNA levels in spinal cord samples derived from mice expressing human WT FUS (hFUS) in either heterozygosity (+/- hFUS) or homozygosity (+/+ hFUS). Error bars are mean ± SEM. N= three mice per group. **J:** Representative agarose gel to investigate CHK1 exon 3 splicing in spinal cord samples derived from mice expressing human WT FUS (hFUS) in heterozygosity (+/- hFUS). The numbers on the gel indicate the PCR cycles after which samples were collected. **K:** Representative agarose gel to investigate CHK1 exon 3 splicing in spinal cord samples derived from mice expressing human WT FUS (hFUS) in homozygosity (+/+ hFUS). The numbers on the gel indicate the PCR cycles after which samples were collected.

**Figure S3 (relative to Figure 3): A:** Quantification of *CHK1* mRNA levels in human motor neuron progenitors (hMNPs) derived from a healthy control (CTRL) or from a sporadic ALS patient (sALS). Error bars are mean ± SEM. N= three independent experiments. **B:** Representative agarose gel to investigate CHK1 exon 3 splicing in hMNPs derived from a healthy control (CTRL) or from a sporadic ALS patient (sALS). **C:** Quantification of the percentage of splicing inclusion (PSI) of CHK1 exon 3 in hMNPs derived from a healthy control (CTRL) or from a sporadic ALS patient (sALS). Error bars are mean ± SEM. N= three independent experiments.

**Figure S4 (relative to Figure 4): A:** Representative western blot showing CHK1 overexpression levels in HeLa cells overexpressing mutant P525L FUS together with a plasmid encoding for FLAG-tagged CHK1 (CHK1-FLAG) or an EV, treated or not with NCS and probed for FLAG. Vinculin was used as loading control. **B:** Representative images of HeLa cells overexpressing mutant P525L FUS and transfected with a non-targeting control siRNA (siCTRL) or with a siRNA targeting CHK1 (siCHK1) and probed for FUS, CHK1 and γH2AX. DNA was counterstained with DAPI. White arrows indicate representative cells with the described phenotype. Scale bar is 20 µM. **C:** Quantification of CHK1 protein nuclear intensity in cells treated as in B. Red dots and error bars are mean ± SEM. N= three independent experiments. At least 50 cells per condition were analysed. **D:** Quantification of γH2AX nuclear intensity in cells treated as in B. Red dots and error bars are mean ± SEM. N= three independent experiments. At least 50 cells per condition were analysed. **E:** Representative western blot showing ASF1A overexpression levels in HeLa cells overexpressing mutant P525L FUS together with a plasmid encoding for FLAG-tagged ASF1A (ASF1A-FLAG) or an EV, treated or not with NCS and probed for FLAG. Vinculin was used as loading control. **F:** Representative images of HeLa cells overexpressing mutant P525L FUS together with ASF1A-FLAG or an EV and probed for FUS, FLAG and γH2AX. DNA was counterstained with DAPI. White arrows indicate representative cells showing the described phenotype. Scale bar is 20 µm. **G:** Quantification of γH2AX nuclear intensity in cells treated as in F. Red dots and error bars are mean ± SEM. N= three independent experiments. At least 30 cells per condition were analysed. **H:** Representative images of HeLa cells overexpressing mutant P525L FUS together with ASF1A-FLAG or an EV, treated or not with NCS and probed for FUS, FLAG and 53BP1. DNA was counterstained with DAPI. White arrows indicate representative cells showing the described phenotype. Scale bar is 20 µm. **I:** Quantification of the number of 53BP1 foci per nucleus in cells treated as in H. Red dots and error bars are mean ± SEM. N= three independent experiments. At least 30 cells per condition were analysed. **J:** Quantification of DROSHA nuclear intensity in HeLa cells treated as in F and probed for FUS, FLAG and DROSHA. Red dots and error bars are mean ± SEM. N= three independent experiments. At least 30 cells per condition were analysed. **K:** Representative western blot showing CHK1 overexpression levels in HeLa cells overexpressing TDP-43 together with CHK1-FLAG or an EV, treated or not with NCS and probed for FLAG. Vinculin was used as loading control. **L:** Quantification of γH2AX nuclear intensity in HeLa cells overexpressing TDP-43 together with CHK1-FLAG or an EV and probed for TDP-43, FLAG and γH2AX. Red dots and error bars are mean ± SEM. N= three independent experiments. At least 30 cells per condition were analysed. **M:** Quantification of the number of 53BP1 foci per nucleus in HeLa cells treated as in K and probed for TDP-43, FLAG and 53BP1. Red dots and error bars are mean ± SEM. N= three independent experiments. At least 30 cells per condition were analysed. **N:** Representative western blot showing ASF1A overexpression levels in HeLa cells overexpressing TDP-43 together with ASF1A-FLAG or an EV, treated or not with NCS and probed for FLAG. Vinculin was used as loading control. **O:** Quantification of γH2AX nuclear intensity in HeLa cells overexpressing TDP-43 together with ASF1A-FLAG or an EV and probed for TDP-43, FLAG and γH2AX. Red dots and error bars are mean ± SEM. N= four independent experiments. At least 30 cells per condition were analysed. **P:** Quantification of the number of 53BP1 foci per nucleus in HeLa cells treated as in N and probed for TDP-43, FLAG and 53BP1. Red dots and error bars are mean ± SEM. N= three independent experiments. At least 30 cells per condition were analysed.

**Figure S5 (related to Figure 5): A:** Representative western blot showing p62 protein levels in HeLa cells overexpressing mutant P525L FUS and treated or not with the autophagy inhibitor bafilomycin A1 (BafA1). H3 was used as loading control. **B:** Quantification of p62 protein levels in cells treated as in A. Error bars are mean ± SEM. N= three independent experiments. **C:** Quantification of the percentage of cells bearing CIs in cells treated as in A. Error bars are mean ± SEM. N= three independent experiments. **D:** Representative images of cells treated as in A and probed for FUS, CHK1 and γH2AX. DNA was counterstained with DAPI. White arrows indicate representative cells showing the described phenotype. Scale bar is 20 µm. **E:** Quantification of CHK1 protein nuclear intensity in cells treated as in D. Red dots and error bars are mean ± SEM. N= three independent experiments. At least 50 cells per condition were analysed. **F:** Quantification of γH2AX nuclear intensity in cells treated as in D. Red dots and error bars are mean ± SEM. N= three independent experiments. At least 50 cells per condition were analysed. **G:** Representative western blot showing GAPDH protein levels in HeLa cells overexpressing mutant P525L FUS and treated or not with the HSC70 inhibitor VER-15508. Vinculin was used as loading control. **H:** Quantification of GAPDH protein levels in cells treated as in G. Error bars are mean ± SEM. N= three independent experiments. **I:** Quantification of the percentage of cells bearing CIs in cells treated as in G. Error bars are mean ± SEM. N= three independent experiments. **J:** Representative images of cells treated as in G and probed for FUS, CHK1 and γH2AX. DNA was counterstained with DAPI. White arrows indicate representative cells showing the phenotype of interest. Scale bar is 20 µm. **K:** Quantification of CHK1 protein nuclear intensity in cells treated as in J. Red dots and error bars are mean ± SEM. N= three independent experiments. At least 50 cells per condition were analysed. **L:** Quantification of γH2AX nuclear intensity in cells treated as in J. Red dots and error bars are mean ± SEM. N= three independent experiments. At least 50 cells per condition were analysed. **M:** Representative western blot showing the accumulation of ubiquitylated proteins in HeLa cells overexpressing mutant P525L FUS and treated or not with the proteasome inhibitor MG132. H3 was used as loading control. **N:** Quantification of ubiquitylated protein levels in HeLa cells treated as in M. Error bars are mean ± SEM. N= three independent experiments. **O:** Quantification of the percentage of cells bearing CIs in HeLa cells treated as in M. Error bars are mean ± SEM. N= three independent experiments. **P:** Representative images of HeLa cells treated or not with the proteasome inhibitor MG132 for the indicated times and probed for CHK1. DNA was counterstained with DAPI. Scale bar is 20 µm. **Q:** Quantification of CHK1 protein nuclear intensity in cells treated as in P. Error bars are mean ± SEM. N= two independent experiments. At least 50 cells per condition were analysed. **R:** Representative western blot showing the accumulation of ubiquitylated proteins in HT-22 cells overexpressing mutant P525L FUS and treated or not with the proteasome inhibitor MG132. H3 was used as loading control. **S:** Quantification of ubiquitylated protein levels in HT-22 cells treated as in R. Error bars are mean ± SEM. N= three independent experiments. **T:** Quantification of the percentage of cells bearing CIs in HT-22 cells treated as in R. Error bars are mean ± SEM. N= three independent experiments.
